# Neurochemical Heterogeneity Among Lateral Hypothalamic Hypocretin/Orexin and Melanin-Concentrating Hormone Neurons Identified Through Single-Cell Gene Expression Analysis

**DOI:** 10.1523/ENEURO.0013-17.2017

**Published:** 2017-09-22

**Authors:** Laura E. Mickelsen, Fredrick W. Kolling, Brock R. Chimileski, Akie Fujita, Carol Norris, Kun Chen, Craig E. Nelson, Alexander C. Jackson

**Affiliations:** 1 Department of Physiology and Neurobiology, University of Connecticut, Storrs, CT 06269; 2 Department of Molecular and Cell Biology, University of Connecticut, Storrs, CT 06269; 3 Department of Biomedical Engineering, University of Connecticut, Storrs, CT 06269; 4 Department of Statistics, University of Connecticut, Storrs, CT 06269; 5 Connecticut Institute for the Brain and Cognitive Sciences, University of Connecticut, Storrs, CT 06269

**Keywords:** cotransmission, hypocretin/orexin, lateral hypothalamic area, melanin-concentrating hormone, neuropeptide, neurotransmitter

## Abstract

The lateral hypothalamic area (LHA) lies at the intersection of multiple neural and humoral systems and orchestrates fundamental aspects of behavior. Two neuronal cell types found in the LHA are defined by their expression of hypocretin/orexin (Hcrt/Ox) and melanin-concentrating hormone (MCH) and are both important regulators of arousal, feeding, and metabolism. Conflicting evidence suggests that these cell populations have a more complex signaling repertoire than previously appreciated, particularly in regard to their coexpression of other neuropeptides and the machinery for the synthesis and release of GABA and glutamate. Here, we undertook a single-cell expression profiling approach to decipher the neurochemical phenotype, and heterogeneity therein, of Hcrt/Ox and MCH neurons. In transgenic mouse lines, we used single-cell quantitative polymerase chain reaction (qPCR) to quantify the expression of 48 key genes, which include neuropeptides, fast neurotransmitter components, and other key markers, which revealed unexpected neurochemical diversity. We found that single MCH and Hcrt/Ox neurons express transcripts for multiple neuropeptides and markers of both excitatory and inhibitory fast neurotransmission. Virtually all MCH and approximately half of the Hcrt/Ox neurons sampled express both the machinery for glutamate release and GABA synthesis in the absence of a vesicular GABA release pathway. Furthermore, we found that this profile is characteristic of a subpopulation of LHA glutamatergic neurons but contrasts with a broad population of LHA GABAergic neurons. Identifying the neurochemical diversity of Hcrt/Ox and MCH neurons will further our understanding of how these populations modulate postsynaptic excitability through multiple signaling mechanisms and coordinate diverse behavioral outputs.

## Significance Statement

The lateral hypothalamic area (LHA) is a key regulator of fundamental behavioral states such as arousal, stress, and reward, and disruption of neural circuits in this region is associated with disorders of sleep, feeding, and motivated behavior. The multifunctional nature of the LHA is attributable to a heterogeneous population of neurons that exhibit significant phenotypic and neurochemical diversity. Here, we sought to resolve aspects of this diversity in two well-studied but incompletely understood LHA neuron populations, defined by their expression of neuropeptides hypocretin/orexin (Hcrt/Ox) and melanin-concentrating hormone (MCH). These efforts lay a foundation for understanding, at a molecular and cellular level, how Hcrt/Ox and MCH neurons coordinate behavioral output and thereby give rise to fundamental innate behavioral states.

## Introduction

The lateral hypothalamic area (LHA) is a powerful modulator of behavioral state through its ability to integrate diverse central and peripheral signals and orchestrate adaptive responses to challenges in the environment. Such functions include the organization of sleep-wake states, feeding, energy balance, and motivated behavior (Berthoud and Münzberg, 2011; Burdakov et al., 2013; Hurley and Johnson, 2014; Brown et al., 2015; Bonnavion et al., 2016; Herrera et al., 2016; Stuber and Wise, 2016), the dysregulation of which are cardinal symptoms of both neuropsychiatric illnesses and metabolic disorders (Wulff et al., 2010; Spiegelhalder et al., 2013; Liu et al., 2014). The LHA thus plays a vital role in coordinating physiologic and behavioral homeostasis to ensure survival (Sternson, 2013).

Underlying the diverse functions of the LHA is a heterogeneous population of neurons, of which only a few have been definitively identified and characterized. Hypocretin/orexin (Hcrt/Ox) and melanin-concentrating hormone (MCH) neurons make up two neuropeptidergic neuronal populations which, despite comprising a relatively small proportion of cells within the LHA, have a profound impact on brain function. Both populations regulate sleep-wake states, but have also been implicated in feeding, metabolism, stress and reward-related behaviors (Burt et al., 2011; Barson et al., 2013; Jones and Hassani, 2013; Brown et al., 2015; Konadhode et al., 2015; Bonnavion et al., 2016; Herrera et al., 2016; Stuber and Wise, 2016)

Hcrt/Ox neurons project widely, serve as key integrators of a diverse set of inputs from both the CNS and periphery, and exert homeostatic control over arousal, feeding, reward and stress. Furthermore, Hcrt/Ox signaling has been implicated in the stabilization of sleep-wake states and its dysfunction has been directly linked to narcolepsy in animal models and humans (Alexandre et al., 2013; Giardino and de Lecea, 2014; Mahler et al., 2014; Sakurai, 2014). Importantly, the electrical activity of Hcrt/Ox neurons is directly correlated with behavioral state, firing at high frequencies during high arousal states and at low frequencies or not at all during low arousal states and sleep (Lee, 2005; Mileykovskiy et al., 2005; Takahashi et al., 2008; Hassani et al., 2009, 2016; González et al., 2016). MCH neurons, in turn, are also unique to the LHA and are implicated in regulating sleep-wake states, as well as feeding and metabolism (Pissios et al., 2006; Monti et al., 2013). Optogenetic experiments show that, whereas activation of Hcrt/Ox neurons promotes wakefulness and behavioral arousal, stimulation of MCH neurons promotes sleep (Adamantidis et al., 2007; Jego et al., 2013; Konadhode et al., 2013; Tsunematsu et al., 2014; Blanco-Centurion et al., 2016; Vetrivelan et al., 2016).

Despite the crucial role of these neurons in maintaining behavioral homeostasis, outstanding questions remain concerning the neurochemical diversity of Hcrt/Ox and MCH neurons, particularly in regard to their coexpression of other neuropeptides and necessary components for the synthesis and release of the fast amino acid neurotransmitters GABA and glutamate. Anatomic data reveal that both populations may be neurochemically heterogeneous in terms of their neuropeptide coexpression and markers for one or more fast neurotransmitters, including machinery for GABA synthesis (GAD65/67), vesicular GABA release (VGAT) and vesicular glutamate release (VGLUT1, VGLUT2, or VGLUT3; Brown et al., 2015; Bonnavion et al., 2016; Stuber and Wise, 2016). A systematic assessment of the neurochemical phenotype of Hcrt/Ox and MCH neurons is lacking owing to the challenge of profiling the expression of many genes in cleanly isolated neurons, which are scattered throughout the LHA and intermingled with other cell populations. Here, we optimized a robust procedure for isolating single, transgenically-labeled Hcrt/Ox and MCH neurons and employed a single-cell quantitative polymerase chain reaction (qPCR) profiling approach to discern their expression of a panel of 48 key genes that encode neuropeptides, fast neurotransmitter components and other key markers. The goal of our study is, through a single-cell gene expression profiling approach, to more fully resolve the neurochemical profile of Hcrt/Ox and MCH neurons, which would shed light on the full extent of their signaling repertoire at postsynaptic targets and inform their role in driving complex, innate behavior.

## Materials and Methods

### Ethics statement

All experiments were performed in accordance with the guidelines described in the National Institutes of Health Guide for the Care and Use of Laboratory Animals and were approved by the Institutional Animal Care and Use Committee of the University of Connecticut.

### Animals

Transgenic mice used to identify Hcrt/Ox neurons expressed enhanced green fluorescent protein (EGFP), under the control of the human prepro-orexin promoter (Sakurai et al., 1999; Yamanaka et al., 2003), referred to here as Ox-EGFP. To identify MCH neurons, we used Tg(*Pmch*-cre)^1Lowl/J^ transgenic mice (Kong et al., 2010; JAX stock 014099, RRID: IMSR_JAX:01499), which was crossed to an enhanced yellow fluorescent protein (EYFP) reporter line, B6.Cg-Gt(ROSA)26Sor^tm3(CAG-EYFP)Hze^/J (Ai3; Madisen et al., 2010; JAX stock 007903, RRID: IMSR_JAX:007903), which selectively expresses EYFP following cre-dependent recombination. The resulting cross is referred to here as *Pmch*-Cre;EYFP. We also used *Slc32a1* ^tm2(cre)Lowl^/J knockin mice (Vong et al., 2011; JAX stock 016962, RRID: IMSR_JAX:016962) to label VGAT-expressing GABAergic neurons and *Slc17a6* ^tm2(cre)Lowl^/J knockin mice (Vong et al., 2011; JAX stock 016963, RRID: IMSR_JAX:016963) to label *Slc17a6*-expressing glutamatergic neurons. Each of these lines were similarly crossed to Ai3 and referred to here as *Vgat*-Cre;EYFP and *Vglut2*-Cre;EYFP, respectively. Wild type mice used for neuroanatomical analysis were C57BL/6 (JAX stock 000664, RRID: IMSR_JAX:000664). All mice were fed *ad libitum* and kept on a 12/12 h light/dark cycle.

### Brain slice preparation for microdissection and single-cell dissociation

Hypothalamic brain slices through the LHA were taken from five Ox-EGFP, 5 *Pmch*-Cre;EYFP, 2 *Vgat*-Cre;EYFP and two *Vglut2*-Cre;EYFP male juvenile mice (postnatal days P21-P23). Brain slice preparation was conducted between 9 and 11 A.M. for the described experiments. Following anesthesia with isofluorane, 225-μm-thick slices were prepared using a vibrating microtome (Campden Instruments model 7000smz-2) in ice-cold, sucrose slicing solution containing 87 mM NaCl, 75 mM sucrose, 25 mM glucose, 25 mM NaHCO_3_, 1.25 mM NaH_2_PO_4_, 2.5 mM KCl, 7.5 mM MgCl_2_, 0.5 mM CaCl_2_, and 5 mM ascorbic acid saturated with 95% CO_2_/5% O_2_. Slices were enzyme-treated for ∼15 min at 34°C using protease XXIII (2.5 mg/ml; Sigma) in a sucrose dissociation solution containing 185 mM sucrose, 10 mM glucose, 30 mM Na_2_SO_4_, 2 mM K_2_SO_4_, 10 mM HEPES, 0.5 mM CaCl_2_ 6 mM MgCl_2_, and 5 mM ascorbic acid, pH 7.4, 320 mOsm. Slices were washed three times with cold dissociation solution then transferred to trypsin inhibitor/bovine serum albumin (BSA; 1 mg/ml; Sigma) in cold sucrose dissociation solution. Typically, three slices were obtained from each animal, that approximately corresponded to mouse brain atlas images representing bregma −1.24, −1.58, and −1.82 mm (Paxinos and Franklin, 2012), which encompassed the region of the LHA caudal to the level of the retrochiasmatic area and rostral to the level of the tuberomammillary nucleus in the posterior hypothalamus. The LHA was microdissected bilaterally from appropriate slices using a 1.0 mm diameter disposable biopsy punch (Ted Pella). Under a dissecting microscope, the tissue punch was placed to optimally excise the region of the LHA from each slice, based on visual identification of key anatomic landmarks: ventral to the mammillothalamic tract (mt), medioventral to the cerebral peduncle (cp), lateral to the dorsomedial nucleus of the hypothalamus, dorsolateral to the ventromedial nucleus of the hypothalamus, and including the fornix in the medioventral quadrant of each punch. Each microdissected brain slice was then photographed and the borders of the tissue punch were subsequently mapped onto mouse brain atlas images (Paxinos and Franklin, 2012). Microdissected tissue punches were kept in sucrose dissociation solution (with trypsin inhibitor/BSA) on ice until trituration. Immediately before dissociation, tissue punches were incubated for ∼10 min in a 37°C water bath, then triturated with a series of small bore fire-polished glass Pasteur pipettes in a volume of 400–600 µl trypsin inhibitor/BSA sucrose dissociation solution. Single-cell suspensions were passed through 60 μm nylon mesh filters to remove any cell aggregates, and kept on ice until fluorescence-activated cell sorting (FACS). to obtain sufficient number of cells for FACS, punches from multiple animals were pooled together for each FACS collection. Ox-EGFP and *Pmch*-Cre;EYFP mice were FACS sorted in two collections each (two to three mice per collection) while *Vgat*-Cre;EYFP and *Vglut2*-Cre;EYFP were sorted in single collections (two mice per collection).

### Fluorescence-activated cell sorting (FACS)

EGFP+ or EYFP+ neurons were sorted on a BD FACSAria II Cell Sorter (UConn Flow Cytometry Facility, Storrs CT, RRID: SCR_012341) equipped with a sapphire 488 nm excitation laser (Coherent). Cells were selected based on scatter and for singlets, then gated on the presence of EGFP/EYFP fluorescence (PE-A vs GFP-A) and sorted in single-cell precision mode with a 10 K threshold into a sterile 96-well plate containing 2.5 µl lysis buffer [0.5% NP-40, 1 U/µl RNAsin+ (Promega), 0.25× pooled TaqMan assays], snap frozen on dry ice, and stored at −80°C until processing for single-cell qPCR. To control for free-RNA contamination in FACS-sorted droplets, calibrite allophycocyanin (APC) beads (Beckton Dickenson) were added to all LHA single-cell suspensions and single beads were sorted based on red/orange fluorescence via FACS using the same gating parameters as single-cells. Beads were snap frozen and underwent qPCR alongside single cells. In initial experiments, single EGFP+ cells were FACS-sorted using the same gating parameters into flat-bottomed wells of a Terasaki plate (Yaron et al., 2014; Tasic et al., 2016) containing 5 μl of 10 mg/ml trypsin inhibitor/BSA in dissociation sucrose and visualized under a fluorescence microscope.

### Single-cell qPCR

Plates of FACS-sorted cells were thawed and a combined lysis/denaturation step was performed by incubation at 70°C/4 min, 4°C/5 min, 2.5 µl reverse transcription master mix was then added to each well [1 µl 5× RT buffer (Promega), 0.6 µl 35 mM MgCl_2_, 0.25 µl Moloney murine leukemia virus (Promega), 0.1 µl 25 mM dNTPs, 0.5 µl H_2_O] and incubated at 37°C/2 min, 42°C/1 min, 50°C/1 s for 40 cycles, then 85°C/5 min, and 4°C hold. Following RT, cDNA was preamplified by adding 2 µl of cDNA from the RT plate to 8 µl of preamp master mix [5 µl TaKaRa premix Taq polymerase (Clontech), 2.5 µl 0.25X TaqMan pool, 0.5 µl H_2_O] and thermocycled at 95°C/3 min, 55°C/2 min, 72°C/2 min, then 95°C/15 s, 60°C/2 min, 72°C/2 min for 16 cycles, and 4°C hold. Amplified cDNA was then diluted 1:50 in nuclease-free H_2_O and this material was used for qPCR against a curated panel of 48 TaqMan Gene Expression Assays (Table 1) on 48.48 dynamic arrays using a Biomark HD system (Fluidigm).

**Table 1.** Panel of 48 genes used to probe the neurochemical profile of Hcrt/Ox and MCH neurons

**Gene name/common protein abbreviation**	**Gene symbol**	**Function**	**TaqMan primer sequence**
Glyceraldehyde-3-phosphate dehydrogenase (GAPDH)	*Gapdh*	Housekeeping	Mm03302249_g1
Hypoxanthine guanine phosphoribosyl transferase (HPRT)	*Hprt*	Housekeeping	Mm03024075_m1
Neuronal nuclei (NeuN)	*Rbfox3*	Neuronal marker	Mm01248781_m1
Microtubule associated protein 2 (MAP2)	*Map2*	Neuronal marker	Mm00485231_m1
Tubulin beta 3 (TuJ1)	*Tubb3*	Neuronal marker	Mm00727586_s1
Glial fibrillary acidic protein (GFAP)	*Gfap*	Glial marker	Mm01253033_m1
S100 calcium binding protein B (S100β)	*S100b*	Glial marker	Mm00485897_m1
Hypocretin/orexin (Hcrt/Ox)	*Hcrt*	Neuropeptide	Mm01964030_s1
Pro-melanin-concentrating hormone (MCH)	*Pmch*	Neuropeptide	Mm01242886_g1
Prodynorphin (DYN)	*Pdyn*	Neuropeptide	Mm00457573_m1
Galanin (GAL)	*Gal*	Neuropeptide	Mm00439056_m1
Neurotensin (NTS)	*Nts*	Neuropeptide	Mm00481140_m1
Nucleobindin 2 (Nesfatin-1 or NUCB2)	*Nucb2*	Neuropeptide	Mm01137144_m1
Cocaine and amphetamine regulatory transcript (CART)	*Cartpt*	Neuropeptide	Mm04210469_m1
Thyrotropin-releasing hormone (TRH)	*Trh*	Neuropeptide	Mm01182425_g1
Prepronociceptin/orphanin FQ (N/OFQ)	*Pnoc*	Neuropeptide	Mm01314909_m1
Corticotropin-releasing hormone (CRF)	*Crh*	Neuropeptide	Mm01293920_s1
Proenkephalin (PENK)	*Penk*	Neuropeptide	Mm01212875_m1
Substance P (SubP)	*Tac1*	Neuropeptide	Mm01166996_m1
Tachykinin2 (TAC2)	*Tac2*	Neuropeptide	Mm01160362_m1
Somatostatin (SST)	*Sst*	Neuropeptide	Mm00436671_m1
Glutamate decarboxylase 2 (GAD65)	*Gad2*	GABA synthesis	Mm00484623_m1
Glutamate decarboxylase 1 (GAD67)	*Gad1*	GABA synthesis	Mm04207432_g1
Vesicular GABA transporter (VGAT)	*Slc32a1*	GABA release	Mm00494138_m1
Vesicular glutamate transporter 1 (VGLUT1)	*Slc17a7*	Glutamate release	Mm00436577_m1
Vesicular glutamate transporter 2 (VGLUT2)	*Slc17a6*	Glutamate release	Mm00499876_m1
Vesicular glutamate transporter 3 (VGLUT3)	*Slc17a8*	Glutamate release	Mm00805413_m1
Vesicle Monoamine Transporter 1 (VMAT1)	*Slc18a1*	Monoamine release	Mm00461868_m1
Vesicle Monoamine Transporter 2 (VMAT2)	*Slc18a2*	Monoamine release	Mm00553058_m1
Glutaminase (GLS)	*Gls*	Glutamate synthesis	Mm01257297_m1
Choline acetyltransferase (ChAT)	*Chat*	Acetylcholine synthesis	Mm01221880_m1
Tyrosine hydroxylase (TH)	*Th*	Catecholamine synthesis	Mm00447557_m1
Histidine decarboxylase (HDC)	*Hdc*	Histamine synthesis	Mm00456104_m1
Tryptophan hydroxylase 1 (TPH1)	*Tph1*	Serotonin synthesis	Mm01202614_m1
Tryptophan hydroxylase 2 (TPH2)	*Tph2*	Serotonin synthesis	Mm00557715_m1
Adenosine deaminase (ADA)	*Ada*	Adenosine metabolism	Mm00545720_m1
Parvalbumin (PV)	*Pvalb*	Calcium-binding protein	Mm00443100_m1
Calretinin (CR)	*Calb2*	Calcium-binding protein	Mm00801461_m1
Calbindin (CALB)	*Calb1*	Calcium-binding protein	Mm00486647_m1
NK2 homeobox 1 (NKX2.1)	*Nkx2-1*	Transcription factor	Mm00447558_m1
LIM homeobox 9 (LHX9)	*Lhx9*	Transcription factor	Mm00495308_m1
Neuronal pentraxin 2 (NPTX2)	*Nptx2*	Synaptic function	Mm00479438_m1
Insulin-like growth factor binding protein 3 (IGFBP3)	*Igfbp3*	Growth factor binding	Mm01187817_m1
CUGBP, Elav-like family member 6 (CELF6)	*Celf6*	RNA binding	Mm01176134_m1
Neuronatin (NNAT)	*Nnat*	Ion channel function	Mm00731416_s1
Leptin receptor (LEPR)	*Lepr*	Receptor	Mm00440181_m1
Ghrelin receptor (GHSR)	*Ghsr*	Receptor	Mm00616415_m1
Melanocortin 4 receptor (MC4-R)	*Mc4r*	Receptor	Mm00457483_s1

### qPCR data analysis

Raw cycle threshold (Ct) values were obtained from the Fluidigm Biomark software and inverted (35-Ct) to generate a log_2_-based scale for gene expression analysis. To eliminate cells with low/no cDNA yield, we filtered our dataset to include only cells within the 95% confidence interval (CI) for *Gapdh* expression after removing cells absent for the transcript. Hierarchical clustering was performed using Ward’s method with complete linkage (Ward, 1963). For principle component analysis (PCA), gene expression was *z* score normalized and processed using the princomp function in R. To examine potential subclusters and/or batch effects, we used both multiple hypothesis testing analysis using custom routines and the fisher.test function in R as well as PCA analysis using the princomp function in R. To quantitatively compare gene expression between Hcrt/Ox and MCH neurons, we performed multiple hypothesis testing on the 48 genes using Fisher’s exact test (Agresti, 1992) to report adjusted *p* values, with the Benjamini-Hochberg procedure (Benjamini and Hochberg, 1995) to control the false discovery rate (FDR) at 5%. All statistical analyses were performed using R (The R Project for Statistical Computing; www.r-project.org, RRID: SCR_001905).

### Statistical power analysis

We performed power analysis to assess whether the numbers of neurons used in this study are adequate to achieve sufficient statistical power in detecting differential gene expression. To this end, we used a simulation in which the sample sizes are fixed at the same values of the real data (Hcrt/Ox: 69; MCH: 89), and the true difference between the two probabilities of expression is set to various levels (0%, 15%, 25%, and 35%). With each simulation, presence/absence data are randomly generated, for which the Fisher’s exact test (Agresti, 1992) was performed at 5% significance level. The simulations were repeated 1000 times under each setting of true probabilities and effect size, and the proportion of times that the test is rejected is then an estimate of the corresponding power. Power analysis via simulation was performed using custom routines in R.

### Fluorescence *in situ* hybridization (FISH)

To prepare tissue sections for FISH, male juvenile (postnatal days P21-P24) wild type C57BL/6 mice were anesthetized with isoflurane, decapitated, and brains were dissected out into ice-cold sucrose. Brains were rapidly frozen on dry ice, embedded in OCT compound and cryosectioned at a thickness of 14 μm onto SuperFrost Plus microscope slides. Sections were fixed with 4% paraformaldehyde (PFA) at 4°C for 15 min, and then dehydrated in 50%, 70%, and 100% ethanol. RNAscope 2.5 Assay (Advanced Cell Diagnostics, ACD, RRID: SCR_012481) was used for all FISH experiments according to manufacturer's protocols (Wang et al., 2012). All RNAscope FISH probes were designed and validated by ACD.

### Imaging and image quantification of FISH data

Confocal images of FISH experiments were obtained using a Leica TSC Sp8 and confocal image files (lif) containing image stacks were loaded into ImageJ (version 2.0.0, NIH, RRID: SCR_003070) and processed to analyze percentage colocalization between mRNA transcripts for various neuropeptide or neurotransmitter components and *Hcrt* or *Pmch*. Cells were marked and categorized using the ImageJ cell counter plugin. Cells showing expression in the orange (TRITC) channel for one of the following mRNA: *Cartpt*, *Nucb2*, *Pdyn*, *Penk*, *Gad1*, *Slc32a1*, or *Slc17a6* were counted and marked. Expression was denoted as binary yes/no dependent on the fulfillment of a defined criteria; the presence of at least five punctate fluorescent dots accompanying a nucleus labeled by 4',6-diamidino-2-phenylindole (DAPI; Vector Laboratories, RRID: AB_2336788). Subsequently, cells showing expression in the green (FITC) channel (for either *Hcrt* or *Pmch*) were similarly marked. Cells that were double marked through this process were identified as cells exhibiting colocalization. This process was repeated for multiple lif files for each FISH experiment, and the total number of colocalized cells versus the total number of *Hcrt-* or *Pmch-*expressing cells was used to create pie charts displaying percentage colocalization, where n values describe the total number of *Hcrt-* or *Pmch-*expressing cells analyzed.

### Immunohistochemistry (IHC)

For IHC analysis, male C57BL/6, Ox-EGFP and *Pmch*-Cre;EYFP mice were transcardially perfused with 10 ml 0.125 M NaCl, then 40 ml of a 1× PBS solution (pH 7.4) containing 4% PFA. Brains were postfixed overnight, then cryoprotected in 30% sucrose for 48 h before being frozen in cold isopentane and stored at −80°C. Frozen brains were cut in coronal sections, 40 μm thick, on a cryostat (Leica CM 3050s). Sections were washed 3 × 5 min in 1× PBS, then washed 3 × 5 min in PBS supplemented with 0.2% Tween 20 (PBST). Slices were incubated in a blocking solution containing PBST supplemented with 2% donkey normal serum for 2 h at room temperature (RT). Slices were then incubated with goat anti-orexin A (1:1000, Santa Cruz Biotechnology, catalog SC-8070, RRID: AB_653610), rabbit anti-MCH (1:1000, Phoenix Pharmaceuticals, H-070-47, RRID: AB_10013632), rabbit anti-CART (1:1000, Phoenix Pharmaceuticals, H-003-61), rabbit anti-nesfatin-1 (NUCB2; 1:1000, Phoenix Pharmaceuticals, H-003-22, RRID: AB_2313672), or mouse anti-GFP (ThermoFisher Scientific, A-11120, RRID: AB_221568). After multiple washes in PBST, the sections were then incubated with appropriate secondary antibody raised in donkey for either Alexa Fluor 488 or Alexa Fluor 594 (1:500, Abcam) in blocking solution for 2 h at RT. Sections were washed 5 × 10 min in PBST and 5 × 10 min in PBS, then mounted onto slides and cover-slipped with Vectashield hardset mounting media with DAPI (Vector Laboratories). Fluorescence images were obtained using a Zeiss Imager M2 (Carl Zeiss MicroImaging) with an Orca-R2 digital CCD camera (Hamamatsu Photonics). Confocal images were obtained using a Leica TSC Sp8. Images were processed using ImageJ (NIH), Photoshop CS (Adobe, RRID: SCR_014199), and Adobe Illustrator CC (Adobe, RRID: SCR_010279) and cell counting was performed on confocal micrographs.

### Brain slice preparation for patch-clamp recording and manual harvest

Coronal brain slices containing the LHA were prepared from male juvenile (P21-P23) Ox-EGFP and *Pmch*-Cre;EYFP mice. Mice were deeply anesthetized with ketamine/xylazine before being transcardially perfused with ice-cold high-sucrose cutting solution before slicing using a vibrating microtome as described previously. Hypothalamic slices containing the LHA (225 μm thick) were then placed in a recovery chamber containing artificial CSF (ACSF) consisting of 125 mM NaCl, 2.5 mM KCl, 25 mM NaHCO_3_, 1.25 mM Na_2_PO_4_, 25 mM glucose, 2 mM CaCl_2_, and 1 mM MgCl_2_, saturated with 95% O_2_/5% CO_2_, and allowed to recover for 45 min at 34–35°C and at least 30 min at RT before recording. Cells were visualized with an upright Olympus BX51 microscope (Olympus America) with infrared differential interference contrast and identified by EGFP expression with epifluorescence illumination. The slice in the recording chamber was superfused with bubbled ACSF at physiological temperature (34–35°C), at which all recordings were performed. All recordings were acquired using a Multiclamp 700B amplifier (Molecular Devices). Whole-cell patch-clamp recordings from EGFP+ Hcrt/Ox neurons were conducted using thin-walled borosilicate glass pipettes (World Precision Instruments) with R_pipette_ values of 2–6 MΩ. Pipettes were backfilled with 1 μl RNase-free internal solution containing 130 mM K-gluconate, 10 mM HEPES, 0.1 mM EGTA, 10 mM NaCl, and 2 mM MgCl_2_ with 20 μg/ml glycogen and 1 U/μl RNase inhibitor made with RNase-free H_2_O. All recordings were conducted using a Multiclamp 700B amplifier (Molecular Devices), digitized at 10 kHz and filtered at 2 kHz using a Digidata 1440 digitizer (Molecular Devices) and were acquired using pClamp10 software (Molecular Devices, RRID:SCR_011323). Data were analyzed using ClampFit (Molecular Devices) and custom procedures in IgorPro (Wavemetrics, RRID: SCR_00325). Only cells with an R_access_ below 15 MΩ were accepted. In current-clamp mode, neurons were subjected to hyperpolarizing and depolarizing step pulses (±40 pA). RNA was collected at the end of recording (typically 3–5 min after initial break-in) by applying light suction until the cell cytoplasm had been aspirated (Citri et al., 2011; Cadwell et al., 2015; Fuzik et al., 2015). The contents of the pipette were expelled using positive pressure into an RNase free PCR tube containing lysis buffer (described above), snap frozen on dry ice and stored at −80°C until further processing for single-cell qPCR.

## Results

### Immunohistochemical validation of Ox-EGFP and *Pmch*-Cre;EYFP transgenic mice

**Figure 1.**
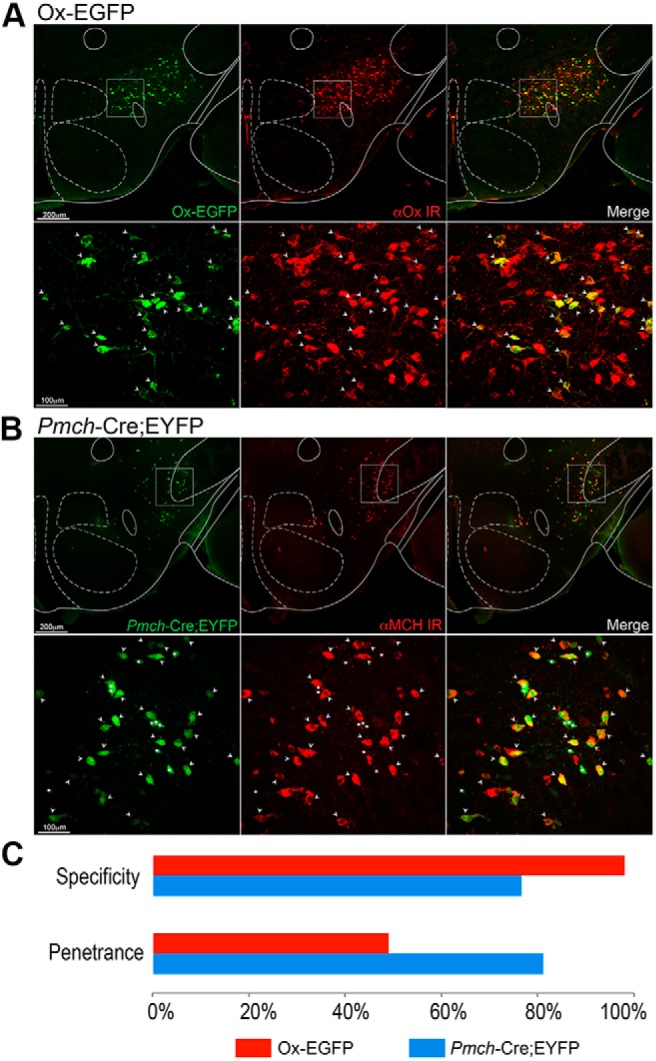
Characterization of Ox-EGFP and *Pmch*-Cre;EYFP mouse lines. ***A***, Fluorescent micrographs (top) of coronal sections of Ox-EGFP mouse brains counterstained for anti-OxA and confocal micrographs of the boxed region (bottom). White arrows indicate co-localization. ***B***, Fluorescent micrographs (top) of coronal sections of *Pmch*-Cre;EYFP mouse brains counterstained for anti-MCH and corresponding confocal micrographs (bottom). White arrows indicate co-localization. ***C***, Bar graph representing specificity and penetrance of Ox-EGFP mice (red, *n* = 3) and *Pmch*-Cre;EYFP (blue, *n* = 3).

To identify labeled Hcrt/Ox and MCH neurons for FACS and for recording in brain slices, we used Ox-EGFP and *Pmch*-Cre;EYFP mice. In both cases, we quantitatively evaluated the fidelity with which each transgenic mouse line expresses EGFP or EYFP in Hcrt/Ox and MCH neurons, respectively, through IHC. To assess specificity of Ox-EGFP mice, we quantified the number of EGFP+ cells which were Hcrt/Ox-immunoreactive (IR) and found the specificity to be ∼98% (*n* = 3 mice). To assess penetrance, we quantified the number of Hcrt/Ox-IR cells that were EGFP+ and found the penetrance to be ∼49% (Fig. 1*C*). We performed a similar analysis using *Pmch*-Cre;EYFP mice (Fig. 1*B*) and found the specificity of this transgenic line to be ∼77% and the penetrance to be ∼81% (*n* = 3 mice; Fig. 1*C*). Thus, we found the Ox-EGFP transgenic mouse to label Hcrt/Ox neurons with high specificity but relatively low penetrance, broadly consistent with the description of the specificity and penetrance of previously described Ox-nLacZ transgenic mice that share the same promoter (Sakurai et al., 1999). In turn, *Pmch*-Cre;EYFP mice were effective in selectively labeling *Pmch*-expressing neurons with reasonably high specificity and penetrance. Although we found penetrance to be in line with the original description of this mouse line (Kong et al., 2010), specificity was less than described. We used both transgenic lines in our subsequent single-cell gene expression profiling and electrophysiological analysis.

### Precise and reproducible microdissection and FACS of Hcrt/Ox and MCH neurons from brain slices

**Figure 2.**
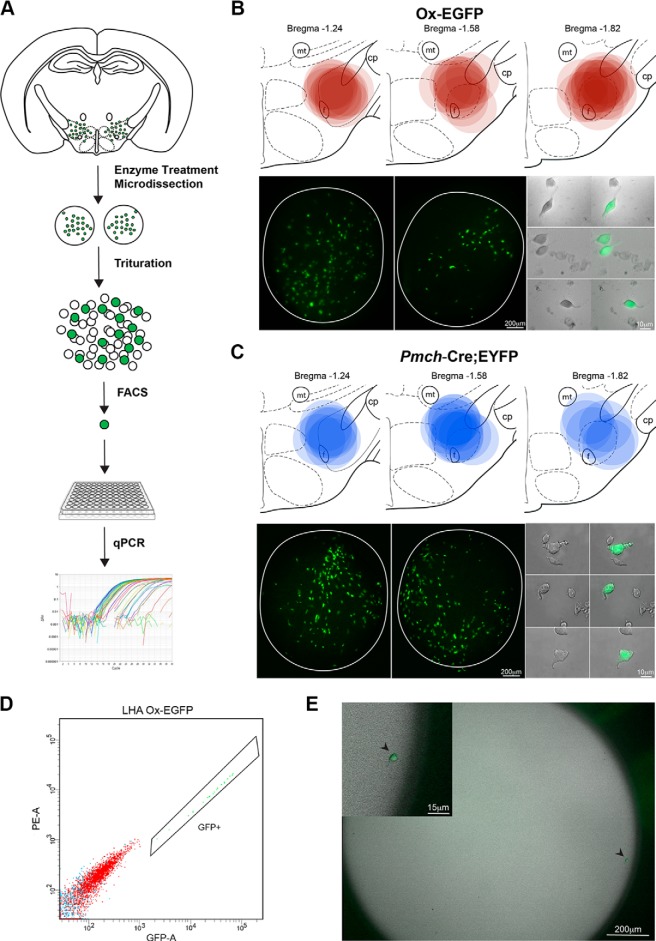
Microdissection of the LHA and isolation of single Ox-EGFP and *Pmch*-Cre;EYFP neurons. ***A***, Schematic representation of microdissection and cell isolation procedure. ***B***, Schematic of the location of tissue punches from Ox-EGFP microdissections mapped onto serial mouse brain atlas images of the hypothalamus (top), representative bilateral tissue punches and isolated EGFP+ and EGFP- neurons (bottom). ***C***, Schematic of tissue punches from *Pmch*-Cre;EYFP microdissections (top), representative tissue punches and isolated EYFP+ and EYFP- neurons (bottom). ***D***, Representative FACS sort of Ox-EGFP neurons showing fluorescence gates for PE-A versus GFP-A showing GFP gate. ***E***, Brightfield and fluorescent micrograph of a representative single Ox-EGFP neuron isolated via FACS in a single well of a Terasaki plate. Higher magnification image of the same Ox-EGFP neuron in the upper left corner inset.

To isolate and capture Hcrt/Ox and MCH neurons for single-cell expression profiling, we optimized a method for the precise and reproducible microdissection of the LHA from coronal brain slices from juvenile mice (P21-P23) and subsequent isolation of single neurons for qPCR via FACS (Fig. 2*A*). In our microdissection, we used a 1 mm diameter circular tissue punch to dissect the LHA from 225-μm-thick coronal brain slices containing the LHA from Ox-EGFP and *Pmch*-Cre;EYFP mice. Following enzyme treatment of whole brain slices, the LHA was visually identified under a dissecting microscope using anatomic landmarks. In all cases, bilateral tissue punches from each slice were subsequently imaged and transposed onto a schematic representation of each of the three representative atlas images to record the accuracy and reproducibility of each microdissection (Fig. 2*B*,*C*). To ensure accuracy of the microdissection in capturing the highest concentration of labeled Hcrt/Ox (Fig. 2*B*) and MCH (Fig. 2*C*) neurons, representative tissue punches were mounted and imaged. Finally, the tissue punches from each preparation were triturated to yield suspensions of healthy LHA neurons with intact fluorescence, shown in representative images (Fig. 2*B*,*C*).

Following gentle trituration and a filtration step to remove aggregates, single-cell suspensions were loaded onto a BD FACSAria II Cell Sorter. To remove debris and minimize the chance of collecting more than a single EGFP+ neuron, a 10K threshold was applied to maximally filter debris in the media. Cells were selected based on scatter, and for singlets, gated on the presence of EGFP/EYFP fluorescence (PE-A vs GFP-A) and sorted into 96-well PCR plates in single-cell purity mode. A representative FACS run, sorting Ox-EGFP neurons under the described conditions, is shown (Fig. 2*D*). To further test the reliability of our FACS configuration to sort only a single EGFP+ neuron in each well, we performed control experiments in which we visualized sorted single cells in 72-well Terasaki plates. As shown in a representative example (Fig. 2*E*), the flat, optically clear bottoms of the wells of Terasaki plates allowed us to visualize the entire field of each well. In initial experiments using Ox-EGFP neurons, we observed only single EGFP+ neurons in each well and failed to detect doublets or any visible debris. Using this method, we were able to successfully sort intact, single EGFP+ or EYFP+ neurons into each well with minimal debris. Further controls for potential contamination were conducted in our subsequent qPCR analysis.

### Panel of 48 key genes used in qPCR analysis of Hcrt/Ox and MCH neurons

To characterize the neurochemical phenotypes of Hcrt/Ox and MCH neurons, we subjected FACS-purified, single Ox-EGFP and *Pmch*-Cre;EYFP neurons to qPCR analysis of the expression of 48 key genes. For this purpose, we used TaqMan Gene Expression Assays on 48.48 dynamic arrays using a microfluidic Biomark HD system (Fluidigm). We developed a custom, curated gene panel (Table 1) comprising a wide variety of genes associated with neuronal function and neurochemical phenotype, selected for their potential ability to serve as positive and negative controls for distinct LHA cell populations based on previous molecular and anatomic characterization. These include housekeeping genes (*Gapdh* and *Hprt*), neuronal markers (*Rbfox3*, *Map2*, and *Tubb3*) and glial markers (*Gfap* and *S100b*) to ensure the analysis of intact, single neurons with minimal glial contamination. We included key neuropeptides known to be expressed in the LHA (*Hcrt*, *Pmch*, *Pdyn*, *Gal*, *Nts*, *Cartpt*, etc.) in addition to others which have not been clearly described in the LHA. Markers of fast amino acid neurotransmission were represented including genes necessary for GABA synthesis (*Gad1* and *Gad2*) and vesicular GABA transport (*Slc32a1*) as well as glutamate vesicular transport (*Slc17a7*, *Slc17a6*, and *Slc17a8*) and metabolism (*Gls*). Genes associated with cholinergic and monoaminergic synthesis and release were also included (*Chat*, *Th*, *Hdc*, *Tph*, *Slc18a2*, etc.) to largely serve as negative controls given the relative dearth of such cell populations in the LHA (Abrahamson and Moore, 2001; Lein et al., 2007). Calcium-binding proteins have shown cell type-specific expression in many other brain regions (Hof et al., 1999), and a *Pvalb*-expressing population of glutamatergic neurons in the LHA has been described (Mészár et al., 2012) and were thus also included (*Pvalb*, *Calb2*, and *Calb1*). Several key transcription factors are known to be relatively specific for LHA cell types, such as *Nkx2.1* for MCH neurons (Croizier et al., 2011) and *Lhx9* for Hcrt/Ox neurons (Shimogori et al., 2010; Dalal et al., 2013; Liu et al., 2015; Sokolowski et al., 2015), and thus serve as important positive controls. To validate findings from previous work (Reti et al., 2002; Crocker et al., 2005; Honda et al., 2009; Shimogori et al., 2010; Dalal et al., 2013; Liu et al., 2015; Sokolowski et al., 2015), we included a number of genes known to be robustly expressed in Hcrt/Ox neurons (*Nptx2*, *Igbp3*, *Celf6*, and *Nnat*), as well as three key receptor genes (*Lepr*, *Ghsr*, and *Mc4r*), implicated in LHA function. Two of these (*Lepr* and *Mc4r*) have been shown to be expressed in overlapping populations of LHA neurons, separate from Hcrt/Ox and MCH neurons (Leinninger et al., 2009; Cui et al., 2012). To ensure only intact neurons with robust gene expression were used for our analysis, all cells collected by FACS were prescreened for the expression of *Gapdh* and outliers (CI = 95%) were removed, resulting in a total of 89 *Pmch*-Cre;EYFP and 70 Ox-EGFP neurons that went on to further qPCR analysis using the full 48-gene panel.

### Single-cell qPCR for transcripts of 48 key genes in Hcrt/Ox and MCH neurons

Following single-cell qPCR, with an output of inverted Ct values plotted on a log_2_-based scale, we first asked what proportion of neurons sorted from Ox-EGFP mice expressed *Hcrt*, and what proportion of *Pmch*-Cre;EYFP neurons expressed *Pmch*. We found that virtually every labeled and sorted Ox-EGFP neuron (98.6%; 69/70) that passed our *Gapdh* prescreen, robustly expressed *Hcrt* (the 1 *Hcrt*- cell was removed from the dataset), and every labeled and sorted *Pmch*-Cre;EYFP neuron (100%; 89/89) expressed *Pmch*, although we observed variability in *Pmch* expression levels. Nevertheless, these data indicate that our dissociation and FACS-sorting procedure, using Ox-EGFP and *Pmch*-Cre;EYFP transgenic lines, is capable of isolating cells that appear to faithfully and selectively express transcripts for their defining neuropeptides.

**Figure 3.**
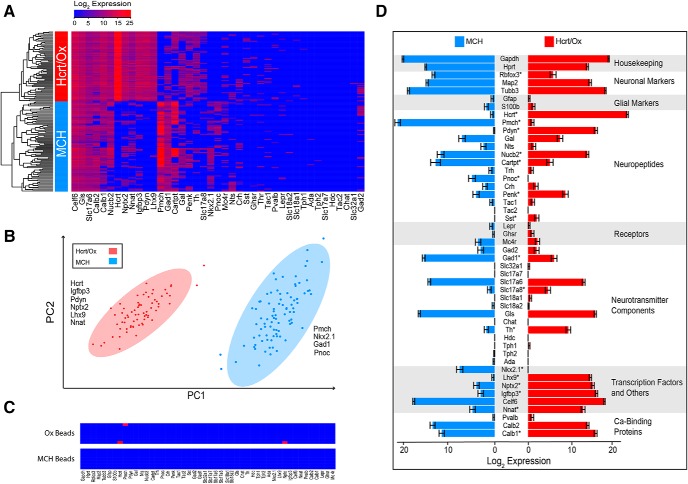
Single-cell qPCR of 48 genes in Hcrt/Ox and MCH neurons. ***A***, Unsupervised hierarchical clustering heatmap of 89 MCH and 69 Hcrt/Ox single cells measured for the expression of 41 genes by qPCR. Genes not represented in this heatmap are housekeeping genes, neuronal and glial markers (*Gapdh*, *Hprt*, *Rbfox3*, *Map2*, *Tubb3*, *Gfap*, and *S100b*). Heatmap colors depict expression levels on a log_2_ scale from low (blue) to high (red). ***B***, Principal component analysis (PCA) using the same cells and genes as in ***A***. Ellipses denote 95% coverage for the populations indicated. Gene names represent transcripts enriched in each population based on the top loading scores from the first principal component. ***C***, Manually ordered heatmap showing 48 gene qPCR of APC bead negative controls from Hcrt/Ox and MCH cell preparations. ***D***, Bar chart of averaged log_2_ expression values for 48 genes in Hcrt/Ox and MCH cells organized by molecular function. Error bars represent standard error of the mean (s.e.m.) for each sample/gene. Asterisks (*) denote statistically significant proportional difference using Fisher’s exact test, adjusted *p* < 0.05 (from Table 2) and the Benjamini-Hochberg procedure to control false discovery rate (FDR) at 5%. In all panels, red denotes Hcrt/Ox neurons and blue denotes MCH neurons.

We then asked whether the gene expression profiles of Hcrt/Ox and MCH neurons are distinct or overlapping based on our 48-gene panel. To do this, we performed unsupervised hierarchical clustering using Ward’s method with complete linkage (Fig. 3*A*). We found that Hcrt/Ox and MCH neurons separated into entirely distinct clusters corresponding to labeled cells isolated from Ox-EGFP and *Pmch*-Cre;EYFP mice as shown in the combined heatmap of gene expression in the two populations (Fig. 3*A*). Genes not represented in this heatmap are housekeeping genes, neuronal and glial markers (Table 1) as well as other genes that are virtually undetectable in both cell populations (*Chat*, *Hdc*, *Slc17a7* and *Tac2*). Genes in this analysis fall into three distinct categories: uniformly expressed in most if not all single cells (e.g., *Celf6*, *Gls*, *Calb1*, *Calb2*, etc.), low/undetectable expression (e.g., *Tph1*, *Tph2*, *Ada*, etc.) or differentially expressed between the two populations (e.g., *Hcrt*, *Pmch*, *Lhx9*, *Nkx2.1*, etc.). Subsequent sections will focus specifically on these differentially expressed genes and their contribution to Hcrt/Ox and MCH neurochemical identity.

To formally determine which genes differentiate the Hcrt/Ox and MCH neuronal populations based on our 48-gene panel, we performed principal component analysis (PCA) and examined the loadings of genes on the PC axes. In our PCA of the single-cell qPCR data, Hcrt/Ox and MCH neurons separate clearly along the first principal component (PC1) into distinct clusters on the basis of several key markers (Fig. 3*B*). The variance captured in PC1 accounts for 26% of the total variance in the dataset. This is in contrast to PC2, which largely captures the within-group variation in Hcrt/Ox and MCH cells, which only captures 8.5% of the total variance. Ellipses represent 95% coverage for each population. Not surprisingly, *Pmch* and *Hcrt* are the most discriminatory markers that differentiate each cluster from one another and show mutually exclusive expression patterns, further confirming that these two populations are nonoverlapping. Hcrt/Ox neurons are further distinguished by the expression of key discriminatory genes (*Igfbp3*, *Pdyn*, *Nptx2*, *Lhx9*, and *Nnat*), confirming previous molecular and anatomic characterizations of Hcrt/Ox neurons (Reti et al., 2002; Crocker et al., 2005; Honda et al., 2009; Shimogori et al., 2010; Dalal et al., 2013; Liu et al., 2015; Sokolowski et al., 2015). However, many of these same markers were also expressed in MCH neurons to varying extents. In particular, we found that *Celf6*, a gene that encodes an RNA-binding protein implicated in autism-related behavior (Dougherty et al., 2013) and found to be widely expressed in neuromodulatory cell types throughout the mouse brain (Maloney et al., 2016), is expressed in all Hcrt/Ox and MCH neurons. In our PCA analysis, we found that MCH neurons are defined by the expression of other discriminatory genes (*Nkx2.1*, *Gad1*, and *Pnoc*). The highly specific expression of *Nkx2.1* in MCH neurons is consistent with previous molecular and anatomic work (Croizier et al., 2011). We note that using PCA analysis, we found that neither Hcrt/Ox nor MCH populations form clear subclusters attributable to the expression of specific discriminatory genes, implying a lack of discrete subpopulations, at least based on our limited 48-gene panel.

We next addressed two potential confounds in the interpretation of our data. One is whether we have sufficient statistical power to quantitatively evaluate differential gene expression between Hcrt/Ox and MCH neurons. Another is whether there are systematic batch effects within each population. To address the first point, we performed power analysis via simulation (see Materials and Methods) to determine if the numbers of neurons we collected for each population are adequate to achieve sufficient statistical power. We found that with the sample sizes of each population (69 and 89) used in this study, Fisher’s exact test (Agresti, 1992) has adequate power (>90%) to detect differential gene expression between the two types of neurons for even moderate effect sizes.

To address the issue of batch effects, we used two complementary approaches. First, we used multiple hypothesis testing to examine whether there is a systematic difference between the two batches within each condition. Specifically, for each population, and for each of the 48 genes, we used Fisher’s exact test (Agresti, 1992) to assess whether the probability of gene expression is the same in the two batches. The Benjamini-Hochberg procedure (Benjamini and Hochberg, 1995) was used to control the FDR at 5%. For Hcrt/Ox neurons, only one gene (*Rbfox3*) shows significant difference among the two batches when the FDR is controlled at 5%. For MCH neurons, three genes (*Gal*, *Trh*, and *Nnat*) show significant difference among the two batches when the FDR is controlled at 5%. Therefore, we found minimal systematic difference between two batches of data for each population. We also performed PCA analysis of batch-effects within each population and found that cells from different collections share nearly identical gene expression patterns and these differences do not contribute to the differences between the Hcrt/Ox and MCH neuron populations.

Finally, another potential confound in our single-cell analysis is the possibility that the droplet of media that accompanies FACS-sorted single cells is a source of contaminating, ambient mRNA from ruptured cells in the cell suspension. To address this possibility, we added fluorescent APC beads to the single-cell suspensions from each population and sorted single beads using parameters described above. Single sorted beads, eight from each condition, were subjected to qPCR alongside our cell samples. Beads sorted from *Pmch*-Cre;EYFP preparations yielded undetectable levels of any of the 48 genes in our panel. Among beads sorted from Ox-EGFP preparations, a single bead contained detectable levels of *Hcrt* and *Nptx2* while another bead contained detectable levels of *Pmch*. These data demonstrate that media contamination is unlikely to contribute to the patterns of gene expression we observed in our single-cell qPCR analysis.

### Differential gene expression between Hcrt/Ox and MCH neurons

Having demonstrated that Hcrt/Ox and MCH neurons are transcriptionally distinct on the basis of our 48-gene panel, we averaged the single-cell expression values from each population to more clearly describe differential gene expression (Fig. 3*D*). Aside from housekeeping genes (*Gapdh* and *Hprt*), Hcrt/Ox and MCH neurons show robust expression of neuronal markers (*Tubb3*, *NeuN*, and *Map2*) and minimal expression of glial markers (*Gfap* and *S100b*), further demonstrating that our capture of single, labeled neurons was largely free of glial contamination. Other broad categories of genes (neuropeptides, receptors, neurotransmitter components etc.) exhibited striking patterns of both common and differential gene expression. To complement average expression level, we quantified proportional difference in gene expression using Fisher’s exact test (Agresti, 1992) to examine whether the probability of gene expression is different among the two populations, while the Benjamini-Hochberg procedure (Benjamini and Hochberg, 1995) was used to control the FDR at 5%. Table 2 shows all 48 genes in descending order of proportional difference, with significance level indicated as adjusted *p* value. Differential gene expression will be addressed in more detail in the subsequent discussion of the coexpression of neuropeptides (Figs. 5, 6) and fast neurotransmitter components (Figs. 7, 8). Taken together, our single-cell qPCR analysis thus indicates that our procedure for isolating single, intact, FACS-sorted Hcrt/Ox and MCH neurons from two transgenically-labeled mouse lines is selective and robust, with minimal contamination by glial cells and ambient transcripts in the media.

**Table 2.** Proportional difference in expression of all 48 genes in Hcrt/Ox and MCH neurons

**Gene symbol**	**Proportion in MCH neurons**	**Proportion in Hcrt/Ox neurons**	**Absolute proportional difference (%)**	**Adjusted *p* value**
*Pdyn*	1/89	69/69	99.0	5.12E-43*
*Lhx9*	3/89	69/69	97.0	2.18E-40*
*Hcrt*	5/89	69/69	94.0	3.93E-38*
*Pmch*	89/89	6/69	91.0	1.59E-36*
*Igfbp3*	20/89	69/69	78.0	6.90E-26*
*Th*	14/89	60/69	71.0	1.84E-19*
*Nptx2*	29/89	69/69	67.0	7.82E-21*
*Nkx2.1*	54/89	0/69	61.0	9.11E-18*
*Nnat*	39/89	68/69	55.0	1.78E-14*
*Penk*	26/89	53/69	48.0	1.43E-08*
*Rbfox3*	86/89	37/69	43.0	1.81E-10*
*Gad1*	87/89	39/69	41.0	1.87E-10*
*Slc17a8*	8/89	33/69	39.0	1.30E-07*
*Pnoc*	31/89	0/69	35.0	5.08E-09*
*Nucb2*	68/89	69/69	24.0	6.23E-06*
*Cartpt*	59/89	29/69	24.0	9.39E-03*
*Sst*	0/89	14/69	20.0	1.21E-05*
*Gal*	49/89	49/69	16.0	>0.05
*Calb1*	76/89	69/69	15.0	1.70E-03*
*Calb2*	80/89	68/69	9.0	>0.05
*Ghsr*	2/89	7/69	8.0	>0.05
*Mc4r*	27/89	16/69	7.0	>0.05
*Slc18a1*	0/89	4/69	6.0	>0.05
*Pvalb*	2/89	6/69	6.0	>0.05
*Nts*	16/89	8/69	6.0	>0.05
*Tac1*	6/89	8/69	5.0	>0.05
*Gad2*	19/89	11/69	5.0	>0.05
*Tph1*	0/89	2/69	3.0	>0.05
*Slc17a6*	87/89	69/69	2.0	>0.05
*Ada*	2/89	0/69	2.0	>0.05
*Lepr*	6/89	3/69	2.0	>0.05
*Crh*	16/89	11/69	2.0	>0.05
*S100b*	16/89	11/69	2.0	>0.05
*Gfap*	4/89	2/69	2.0	>0.05
*Slc32a1*	0/89	1/69	1.0	>0.05
*Map2*	88/89	69/69	1.0	>0.05
*Tph2*	1/89	0/69	1.0	>0.05
*Trh*	7/89	6/69	1.0	>0.05
*Slc18a2*	2/89	1/69	1.0	>0.05
*Hprt*	89/89	69/69	0.0	>0.05
*Gapdh*	89/89	69/69	0.0	>0.05
*Slc17a7*	0/89	0/69	0.0	>0.05
*Hdc*	0/89	0/69	0.0	>0.05
*Tac2*	0/89	0/69	0.0	>0.05
*Tubb3*	89/89	69/69	0.0	>0.05
*Gls*	89/89	69/69	0.0	>0.05
*Chat*	0/89	0/69	0.0	>0.05
*Celf6*	89/89	69/69	0.0	>0.05

Asterisks (*) denote significant difference (Fisher’s exact text and Benjamini-Hochberg procedure to control FDR at 5%).

### Comparison of single-cell qPCR analysis of Hcrt/Ox neurons with bulk TRAP/microarray data

**Figure 4.**
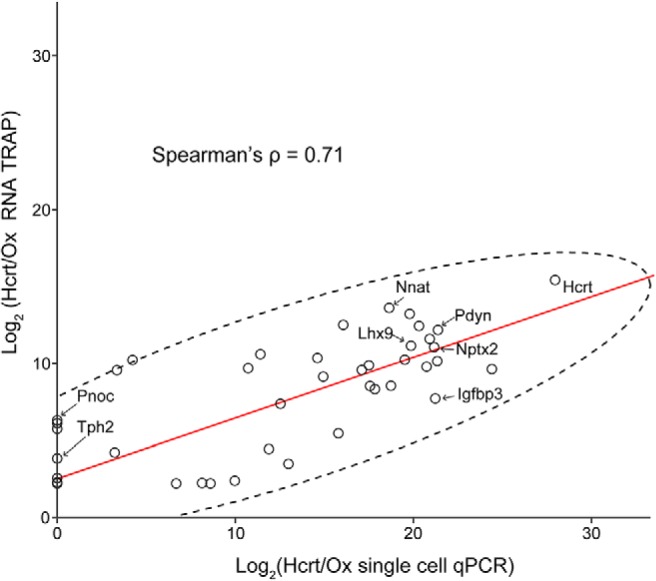
Comparison of single-cell qPCR data to previously published RNA-TRAP dataset. Scatterplot showing expression of all 41 genes measured in both our single-cell qPCR dataset (*x*-axis) and in a previously published bulk RNA TRAP experiment (*y*-axis; Dalal et al., 2013). The two datasets show strong correlation (Spearman's ρ = 0.71; *p* = 1.2 × 10^−8^), the red line depicts the Spearman's correlation, dotted ellipse denotes 95% coverage of the data points.

As further validation of the pattern of gene expression that we observed in our single-cell qPCR analysis of Hcrt/Ox neurons, we compared our dataset with a previously described bulk transcriptomic analysis of Hcrt/Ox neurons (Dalal et al., 2013). In this work, Dalal and coauthors generated a novel transgenic mouse line (Hcrt::eGFP-RpL10a), isolated ribosome-bound transcripts from homogenized diencephalon through translating ribosome affinity purification (TRAP) methodology, followed by systematic translational profiling through microarrays. Using this published data, we identified 41 genes that were common to both their dataset and our gene panel and directly compared their gene expression values with our averaged single-cell qPCR data (Fig. 4). We found a strong positive linear correlation between these two datasets, with a Spearman’s correlation coefficient ρ = 0.71 (*p* = 1.2 × 10^−8^). Among the most strongly correlated genes were *Hcrt*, *Pdyn*, *Nptx2*, *Igfbp3*, *Lhx9*, and *Nnat*, all known to be enriched in Hcrt/Ox neurons from Dalal et al. (2013) and from previous work (Reti et al., 2002; Crocker et al., 2005; Honda et al., 2009; Shimogori et al., 2010; Liu et al., 2015; Sokolowski et al., 2015). This robust correlation of known discriminatory markers, observed using two independent methodologies and transgenic reagents, is additional supportive evidence that our single-cell qPCR approach is capable of capturing pure populations of rare and intermingled neuropeptidergic neurons within the LHA.

### Neuropeptide and receptor expression varies widely in specificity and abundance among Hcrt/Ox and MCH neuron populations

**Figure 5.**
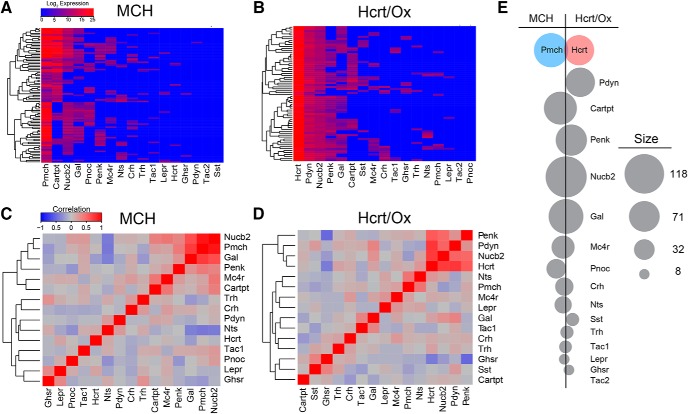
Analysis of neuropeptide and receptor coexpression patterns. ***A***, ***B***, Unsupervised hierarchical clustering heatmap of neuropeptide gene expression in MCH (***A***) and Hcrt/Ox (***B***) neurons. ***C***, ***D***, Clustered heatmap of pairwise Pearson correlations between neuropeptides in MCH (***C***) and Hcrt/Ox (***D***) neurons. ***E***, Bubble plot depicts the net difference in expression frequency for the neuropeptides indicated. Neuropeptides with a higher frequency of detection in MCH cells are shown to the left of the black line, higher frequency of detection in Hcrt/Ox cells to the right. Area of the circles represents the total number of cells from both populations expressing a given neuropeptide.

We next sought to determine how the expression of 14 neuropeptides (*Hcrt*, *Pmch*, *Cartpt*, *Nucb2*, *Gal*, *Pnoc*, *Penk*, *Nts*, *Crh*, *Tac1*, *Tac2*, *Sst*, *Trh*, and *Pdyn*) and three key peptide receptors (*Lepr*, *Ghsr*, and *Mc4r*) was distributed between MCH and Hcrt/Ox neuron populations (Fig. 5; Table 2). We asked whether these 17 neuropeptide/receptor markers exhibited mutually exclusive expression patterns or were common to both populations. For both MCH and Hcrt/Ox neurons, we visualized patterns of coexpression among the individual neuropeptide/receptor markers in hierarchically-clustered heatmaps (Fig. 5*A*,*B*), correlation matrices (Fig. 5*C*,*D*), and bubble plots (Fig. 5*E*).

As described above, we found that every labeled and sorted Ox-EGFP neuron expressed *Hcrt* and every labeled and every sorted *Pmch*-Cre;EYFP neuron expressed *Pmch* to varying degrees (Fig. 5; Table 2). However, we also found that a small number of Hcrt/Ox neurons also expressed *Pmch* (8.7%), and an even smaller number of MCH neurons also expressed *Hcrt* (5.6%; Fig. 5; Table 2). This potential crosstalk is mitigated by our observation of highly selective expression of two key discriminatory transcription factors, *Lhx9* in Hcrt/Ox neurons (100% in Hcrt/Ox neurons; 3.4% in MCH neurons) and *Nkx2.1* in MCH neurons (60.1% in MCH neurons; 0% in Hcrt/Ox neurons; Fig. 3*D*; Table 2).

Among MCH neurons, the most extensively coexpressed neuropeptide transcripts, in descending order, were *Nucb2* (76.4%), *Cartpt* (66.3%), *Gal* (55.0%), *Pnoc* (34.8%), and *Penk* (29.2%; Fig. 5; Table 2) reflected in the clustering observed in the correlation matrix (Fig. 5*C*). Neuropeptide transcripts that exhibited particularly low expression levels in MCH neurons include *Sst* (0%), *Tac2* (0%), *Pdyn* (1.1%), and *Trh* (7.9%). Among Hcrt/Ox neurons, the most extensively coexpressed neuropeptide transcripts in our panel were *Pdyn* (100%), *Nucb2* (100%), *Penk* (76.8%), and *Cartpt* (42.0%; Fig. 5; Table 2). Neuropeptide transcripts that exhibited notably low expression levels in Hcrt/Ox neurons include *Tac2* (0%), *Pnoc* (0%), and *Trh* (8.7%).

Neuropeptides/receptors exhibit varying degrees of specificity and abundance within the MCH and Hcrt/Ox populations, summarized in a bubble plot (Fig. 5*E*; Table 2). For example, *Pdyn* is unsurprisingly both highly abundant and highly specific to Hcrt/Ox neurons, consistent with previous work (Chou et al., 2001; Muschamp et al., 2014) and the only neuropeptide transcript to exhibit this profile. Other neuropeptides (*Sst* and *Pnoc*), although much less abundant, also exhibit highly specific differential expression (*Sst* in a small subpopulation of Hcrt/Ox neurons and *Pnoc* in a subpopulation of MCH neurons). Our observation that *Pnoc*, encoding nociceptin/orphanin FQ (N/OFQ), exhibits moderate to low expression in MCH but is undetectable in Hcrt/Ox neurons, contrasts with a previous report showing that N/OFQ-IR is found in most Hcrt/Ox but not MCH neurons in the rat (Maolood and Meister, 2010), but is consistent with a study showing that N/OFQ-IR and Hcrt/Ox-IR neurons are intermingled but separate (Gerashchenko et al., 2011). Other neuropeptides (*Cartpt* and *Penk*) are abundant and show only moderate specificity, with *Cartpt* being moderately specific to MCH neurons, with clear *Cartpt*+ and *Cartpt*- subpopulations (especially prominent in Fig. 5*A*), while *Penk* is moderately specific to Hcrt/Ox neurons. Detailed analyses in rodents demonstrated extensive and selective expression of *Cartpt* in a subpopulation of MCH neurons, with little to no expression in Hcrt/Ox neurons (Broberger, 1999; Vrang et al., 1999; Brischoux et al., 2001; Elias et al., 2001; Cvetkovic et al., 2004). *Penk* expression has been reported in the rat LHA (Abrahamson and Moore, 2001; Horjales-Araujo et al., 2014), although colocalization with Hcrt/Ox and MCH neurons has not been previously shown.

In contrast, other neuropeptide transcripts were either abundant or absent from both populations. *Nucb2* shows significantly higher expression in Hcrt/Ox neurons (Table 2) but is highly abundant in both populations, in contrast to previous work showing strong specificity for MCH neurons (Foo et al., 2008; Fort et al., 2008). *Gal* was also relatively abundant but seemingly nonspecific, contrasting with previous work (Laque et al., 2013). Similarly, other neuropeptide transcripts (*Tac1*, *Crh*, *Nts*, *Trh*) also show little specificity but are much less abundant, while *Tac2* is undetectable in either population. Our failure to detect appreciable *Nts* transcript in Hcrt/Ox neurons contrasts with a previous report showing that a majority of Hcrt/Ox neurons express *Nts* mRNA and NTS-IR (Furutani et al., 2013). Receptor expression in both populations is relatively low; *Mc4r* is expressed in small proportion of both populations with no specificity while both *Ghsr* and *Lepr* are neither abundant nor specific. The low expression of *Mc4r*, *Lepr*, and *Nts* in either population is consistent with MCH and Hcrt/Ox neurons being entirely separate from overlapping populations of largely leptin-sensitive, neuropeptidergic LHA neurons (Leinninger et al., 2009, 2011; Cui et al., 2012; Laque et al., 2013). Taken together, these data suggest that both Hcrt/Ox and MCH neurons exhibit a surprisingly high degree of coexpression with other neuropeptide transcripts, with only a few showing both high abundance and specificity for one population over another.

### *In situ* hybridization and immunohistochemical validation of neuropeptide coexpression in Hcrt/Ox and MCH neurons

**Figure 6.**
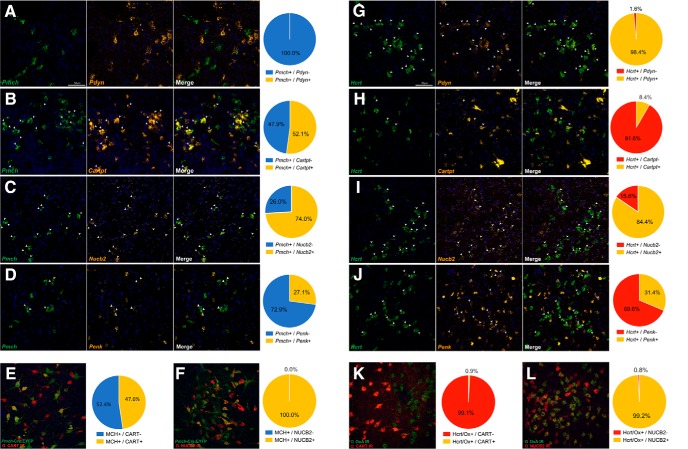
*In situ* hybridization and immunohistochemical validation of neuropeptide expression in Hcrt/Ox and MCH neurons. ***A–D***, Confocal micrographs (40×) of coronal sections from wild type mice and corresponding pie charts representing coexpression of mRNA for *Pmch* and *Pdyn*, *n* = 47 cells (***A***), *Pmch* and *Cartpt*, *n* = 190 cells (***B***), *Pmch* and *Nucb2*, *n* = 77 cells (***C***), and *Pmch* and *Penk*, *n* = 75 cells (***D***); all counterstained with DAPI (blue). White arrows indicate coexpression. ***E***, ***F***, Confocal micrographs (40×) of coronal sections from *Pmch-cre*;EYFP mice counterstained for anti-GFP (green), anti-CART (red), *n* = 134 cells (***E***), and anti-NUCB2 (red), *n* = 134 (***F***); all counterstained for DAPI (blue). ***G–J***, Confocal micrographs (40×) of coronal sections from wild type mice and corresponding pie charts representing coexpression of mRNA for *Hcrt* and *Pdyn*, *n* = 125 cells (***G***), *Hcrt* and *Cartpt*, *n* = 154 cells (***H***), *Hcrt* and *Nucb2*, *n* = 90 cells (***I***), and *Hcrt* and *Penk*, *n* = 169 cells (***J***); all counterstained with DAPI (blue). White arrows indicate coexpression. ***K***, ***L***, Confocal micrographs (40×) of coronal sections from wild type mice counterstained for anti-OxA (green) and anti-CART (red), *n* = 115 (***K***), and anti-NUCB2 (red), *n* = 115 (***M***); all counterstained with DAPI (blue).

To validate the coexpression of neuropeptide mRNA in *Hcrt-* and *Pmch*-expressing neurons, we used dual FISH using the ACD RNAscope 2.5 Manual Assay for four key neuropeptides: *Pdyn*, *Cartpt*, *Penk*, and *Nucb2*, and measured percentage colocalization (Fig. 6*A–D*,*G–J*). Coronal LHA sections taken from juvenile wild type mice were counterstained with FISH probes and imaged using confocal microscopy. We confirmed a lack of colocalization of *Pdyn* mRNA with MCH neurons (0/47 cells; 0%) and extensive colocalization of *Pdyn* mRNA in Hcrt/Ox neurons (123/125 cells; 98.4%; Fig. 6*A*,*G*). We found *Cartpt* mRNA to colocalizes with 52.1% (99/190 cells) of MCH neurons but only 8.4% (13/154 cells) of Hcrt/Ox neurons (Fig. 6*B*,*H*). Colocalization of *Nucb2* mRNA was found in a majority of MCH (57/77 cells; 74.0%) and Hcrt/Ox (76/90 cells; 84.4%) neurons (Fig. 6*C*,*I*). We also observed limited overlap of *Penk* mRNA with both MCH (23/85 cells; 27.1%) and Hcrt/Ox (53/169 cells; 31.4%) neurons (Fig. 6*D*,*J*). We confirmed the presence of CART and nesfatin-1 (NUCB2) in MCH neurons using IHC, where *Pmch-*Cre;EYFP coronal brain slices were sectioned and stained for antibodies against either CART or NUCB2. We observed extensive colocalization of MCH neurons with CART-IR (60/126 cells, 47.6%) and complete colocalization of MCH neurons with NUCB2-IR (134/134 cells, 100.0%; Fig 6*E*,*F*). We similarly confirmed the presence of CART-IR and NUCB2-IR in Hcrt/Ox neurons using wild type coronal slices counterstained for an antibody against orexin A and either CART or NUCB2. We confirmed robust colocalization of NUCB2-IR in Hcrt/Ox neurons (119/120 cells, 99.2%), but did not observe robust CART-IR in Hcrt/Ox neurons (1/115 cells, 0.8%; Fig. 6*K*,*L*). This last observation contrasts with both our qPCR and FISH data and suggests that perhaps Hcrt/Ox neurons may contain *Cartpt* mRNA but little detectable protein. Nevertheless, both our FISH and IHC data are broadly consistent with our single-cell qPCR analysis of *Pdyn*, *Cartpt*, *Nucb2*, and *Penk* in Hcrt/Ox and MCH neurons within the LHA.

### Hcrt/Ox and MCH neurons exhibit markers of both GABAergic and glutamatergic fast neurotransmitter phenotypes

**Figure 7.**
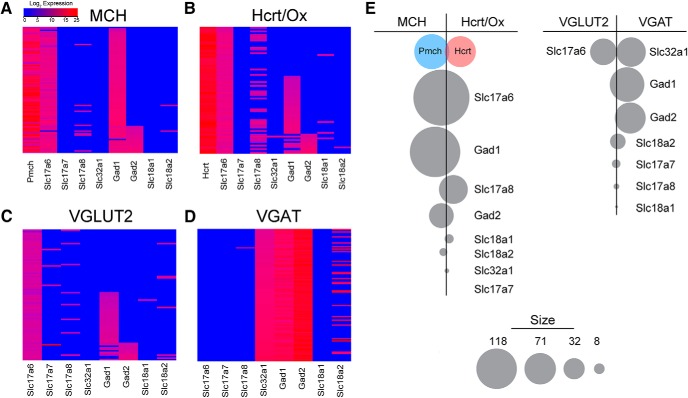
Analysis of fast amino acid neurotransmitter phenotypes. ***A***, ***B***, Manually ordered heatmaps of neurotransmitter expression in MCH (***A***) and Hcrt/Ox (***B***) neurons, arranged by function in glutamatergic or GABAergic release pathways. ***C***, ***D***, Manually ordered heatmaps of neurotransmitter expression in VGLUT2+ (***C***) and VGAT+ (***D***) neurons isolated from *Vglut2*-Cre;EYFP and *Vgat*-Cre;EYFP mice, respectively, arranged by function in glutamatergic or GABAergic release pathways. ***E***, Bubble plot depicts the net difference of detection in expression frequency of the neurotransmitter component indicated. Left, Neurotransmitters with higher frequency of detection in MCH neurons are to the left of the black line, higher frequency in Hcrt/Ox neurons to the right. Right, Neurotransmitters with higher frequency of detection in VGLUT2 neurons are to the left of the black line, higher frequency in VGAT neurons to the right.

While MCH and Hcrt/Ox neurons are widely thought to exhibit GABAergic and glutamatergic phenotypes, respectively, recent evidence suggests greater ambiguity in their fast neurotransmitter phenotype than previously appreciated (Bonnavion et al., 2016). To address this conflicting evidence using our single-cell qPCR dataset, we compared the expression patterns of genes necessary for fast amino acid neurotransmitter synthesis and vesicular transport among MCH (Fig. 7*A*) and Hcrt/Ox neurons (Fig. 7*B*) and summarized their differential expression in the form of bubble plots (Fig. 7*E*).

Consistent with the widely held view that MCH neurons are GABAergic in nature, we found that virtually all MCH neurons express the GABA synthetic enzyme *Gad1* (97.8%) and a smaller subpopulation expresses *Gad2* (21.3%; Fig. 7; Table 2). However, none of the MCH neurons exhibited detectable expression of the vesicular GABA transporter *Slc32a1* (0%) This suggests that while these cells may be capable of GABA synthesis, they lack the machinery for its vesicular packaging and release through canonical pathways. In contrast, the vesicular glutamate transporter 2 (*Slc17a6*) was expressed in virtually every MCH neuron (97.8%) with a very small subpopulation of neurons expressing the vesicular glutamate transporter 3, *Slc17a8* (9.0%; Fig. 7*A*), suggesting a functional glutamatergic phenotype. Similarly, we found that every Hcrt/Ox neuron expressed *Slc17a6* (100%) approximately half of which also express *Slc17a8* (47.8%; Fig. 7; Table 2), consistent with evidence showing that these cells are glutamatergic. Surprisingly, over half of the Hcrt/Ox neurons we sampled expressed *Gad1* (56.5%) and a smaller subpopulation expressed *Gad2* (15.9%). As with MCH neurons, we found that *Slc32a1* expression was virtually undetectable in Hcrt/Ox neurons (1.5%). Finally, *Slc17a7* expression was undetectable in either population. Given the abundant expression of *Gad1* in both populations, and failure to detect *Slc32a1*, we asked whether Hcrt/Ox and MCH neurons expressed the vesicular monoamine transporters *Slc18a1* and *Slc18a2*, the latter of which has been shown to be capable of the transport and vesicular release of GABA through a noncanonical pathway (Tritsch et al., 2012). We found, however, that only a very small fraction of MCH (2.5%) and Hcrt/Ox (1.5%) neurons express *Slc18a2* transcript at detectable levels.

Having observed the surprising result that these two populations of neuropeptidergic neurons in the LHA uniformly and abundantly express the machinery for both glutamate release (*Slc17a6*) and GABA synthesis (*Gad1*), we asked if this pattern of coexpression is a common feature of glutamatergic neurons in the LHA. To this end, we performed an identical single-cell qPCR analysis of *Slc17a6*-expressing LHA neurons from *Vglut2*-Cre;EYFP mice. As these mutant mice label cells with both transient developmental and stable expression of *Slc17a6*, we isolated 117 single labeled neurons from microdissected slices that passed *Gapdh* prescreening and confined our analysis to the 86 neurons expressing the *Slc17a6* transcript (73.5%). Of these cells, none expressed *Slc32a1* (0%; 0/86; Fig. 7*C*). However, exactly half of these *Slc17a6*-expressing neurons expressed *Gad1* (50%; 43/86) along with another small, partially overlapping population expressing *Gad2* (14.0%; 12/86). Although the sample size is limited, these data suggest that approximately half of *Slc17a6*-expressing, putative glutamatergic neurons in the LHA are potentially capable of GABA synthesis.

Finally, considering that in our analysis of Hcrt/Ox, MCH and glutamatergic neurons, we did not detect expression of *Slc32a1*, we asked whether this was the result of a true lack of expression or the failure of our probe to detect *Slc32a1* expression. Furthermore, we wished to confirm the neurotransmitter phenotype of canonical GABAergic neurons in the LHA (i.e., those capable of both the synthesis and vesicular release of GABA). For this purpose, we performed a single-cell qPCR analysis of *Slc32a1*-expressing neurons from the LHA of *Vgat*-Cre;EYFP mice. Similar to the *Vglut2*-expressing cells collected above, we isolated 212 single neurons and focused our analysis on the 118 cells expressing the *Slc32a1* transcript (55.7%). We found that virtually none of these cells expressed vesicular glutamate transporters (*Slc17a6*: 0%; 0/118, *Slc17a7*: 0%; 0/118, *Slc17a8*: 0.8%; 1/118). However, all *Slc32a1*-expressing neurons robustly and uniformly expressed the triad of canonical markers of GABAergic phenotype (*Slc32a1*: 100%; 118/118, *Gad1*: 100%; 118/118, *Gad2*: 100%; 118/118). These data confirm that we can detect *Slc32a1* in GABAergic neurons and that LHA GABAergic neurons uniformly and abundantly express markers of canonical GABA synthesis and release (*Slc32a1*, *Gad1*, and *Gad2*).

### *In situ* hybridization validation of fast amino acid neurotransmitter marker expression in Hcrt/Ox and MCH neurons

**Figure 8.**
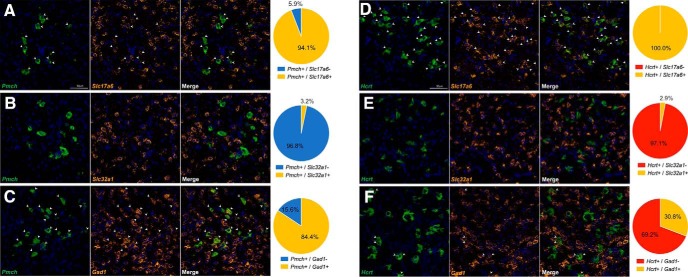
*In situ* hybridization validation of fast amino acid neurotransmitter expression in Hcrt/Ox and MCH neurons. ***A–C***, Confocal micrographs (40×) of coronal sections from wild type mice and corresponding pie charts representing coexpression of mRNA for *Pmch* and *Slc17a6*, *n* = 51 cells (***A***), *Pmch* and *Slc32a1*, *n* = 63 cells (***B***), and *Pmch* and *Gad1*, *n* = 224 cells (***C***); all sections counterstained with DAPI (blue). ***D–F***, Confocal micrographs (40×) of coronal sections from wild type mice and corresponding pie charts representing coexpression of mRNA for *Hcrt* and *Slc17a6*, *n* = 45 cells (***D***), *Hcrt* and *Slc32a1*, *n* = 170 cells (***E***), and *Hcrt* and *Gad1*, *n* = 237 (***F***); all sections counterstained with DAPI (blue). White arrows indicate coexpression.

To validate the expression of *Slc17a6* and *Gad1*, as well as the absence of *Slc32a1*, in Hcrt/Ox and MCH neurons we again used FISH analysis (Fig. 8*A–F*). We confirmed extensive overlap of MCH (48/51 cells; 94.1%) and complete overlap of Hcrt/Ox (45/45 cells; 100%) neurons with *Slc17a6* mRNA (Fig. 8*A*,*D*), with minimal colocalization of *Slc32a1* mRNA with MCH neurons (2/63 cells; 3.2%) and Hcrt/Ox (5/170 cells; 2.9%; Fig. 8*B*,*E*). Furthermore, we found that 84.4% (189/224) of MCH neurons colocalized with *Gad1* mRNA (Fig. 8*C*) while approximately 30.8% (73/237) of Hcrt/Ox neurons contained *Gad1* mRNA (Fig. 8*F*). These data are broadly consistent with our single-cell qPCR analysis of *Slc17a6*, *Gad1*, and *Slc32a1* in Hcrt/Ox and MCH neurons within the LHA.

### Comparing electrophysiological diversity with neurochemical diversity in Hcrt/Ox neurons

Finally, we asked whether the neurochemical heterogeneity that we identified among Hcrt/Ox and MCH neurons correlates with known functional diversity. To approach this question, we turned to the well-characterized intrinsic membrane properties of Hcrt/Ox neurons.

Previous work in brain slices from Ox-EGFP mice has shown that, on the basis of key intrinsic membrane properties, morphologic characteristics and measures of synaptic input, Hcrt/Ox neurons may be parsed into two functional subpopulations (Schöne et al., 2011). In particular, Schöne and coauthors found that the signature of one population (“D-type”) is a depolarizing rebound in firing following a hyperpolarizing step in current-clamp mode. Another population (“H-type”), in turn, exhibited a characteristic long latency to firing following the same hyperpolarizing step as a result of prominent expression of A-type potassium current (Burdakov et al., 2004; Schöne et al., 2011). Neither population was shown to segregate on the basis of their projection targets (González et al., 2012). We asked whether we could (1) recapitulate this broad categorization; (2) harvest cytoplasm from recorded cells for qPCR analysis using the same 48-gene panel and correlate gene expression with FACS-sorted cells; and (3) determine if D-type and H-type signatures segregated between neurochemically distinct Hcrt/Ox neurons at the single-cell level. For example, can *Gad1*+ and *Gad1*- Hcrt/Ox neurons be identified on the basis of key electrical signatures?

**Figure 9.**
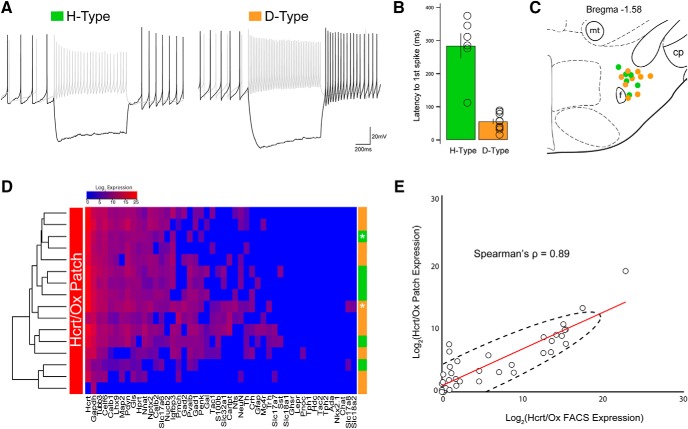
Manual (patch) harvest of mRNA from Hcrt/Ox neurons and correlation between neurochemcial phenotype and membrane properties. ***A***, Representative current-clamp recordings of two Hcrt/Ox neurons responding to +40 pA (gray) and −40 pA (black) current injections, identifying H-type (left) and D-type (right) signatures. In all panels, green denotes H-type while orange denotes D-type. ***B***, Latency to first spike after a 1 s hyperpolarizing step of −120 pA, threshold for H-type set at 100 ms (*n* = 16 cells; 6 H-type, 10 D-type). ***C***, Schematic of a representative LHA slice with the approximate location of recorded H-type and D-type Hcrt/Ox neurons. ***D***, Unsupervised hierarchical clustering heatmap of 16 Hcrt/Ox neurons measured for the expression of 48 genes by qPCR, along with their electrical signature. Asterisks indicate the two individual cells corresponding to traces in ***A***. ***E***, Scatterplot showing expression of all 48 genes measured in both our single-cell Hcrt/Ox qPCR dataset (*x*-axis) and patch harvested Hcrt/Ox qPCR dataset (*y*-axis). The two datasets show strong correlation (Spearman's ρ = 0.89; *p* = 2.2 × 10^−16^).

We obtained slices from Ox-EGFP mice and performed patch-clamp recordings from 16 EGFP+ neurons. From current-clamp recordings, we found that 62.5% (10/16) Hcrt/Ox neurons exhibited a depolarizing postinhibitory rebound following a 1 s hyperpolarizing step, consistent with the D-type signature. In turn, 37.5% (6/16) Hcrt/Ox neurons exhibited a latency (>100 ms) to firing following the hyperpolarizing step, consistent with the H-type signature (Fig. 9*A*,*B*). We also noted the location of each recorded cell and mapped them onto an atlas image of the LHA (Fig. 9*C*). We then harvested cytoplasm from each of these neurons through the recording pipette and performed single-cell qPCR analysis using our 48-gene panel, in an otherwise identical manner to FACS-sorted cells. We found that 93.7% (15/16) of these Hcrt/Ox neurons expressed *Hcrt* in addition to other key discriminatory genes known to be enriched in Hcrt/Ox neurons including *Lhx9*, *Nptx2*, *Pdyn*, and *Nnat*. In performing unsupervised hierarchical clustering of the gene expression data, we failed to detect a clear correlation between neurochemically diverse Hcrt/Ox neurons and electrophysiological signatures (Fig. 9*D*). Despite this, when we compared gene expression patterns between recorded cells and FACS-sorted cells, we found a strong, positive linear correlation with a Spearman's correlation coefficient ρ = 0.89 (*p* = 2.2 × 10^−16^; Fig. 9*E*). Taken together, these data suggest that although distinct electrical signatures of Hcrt/Ox neurons may not readily track with the known markers of neurochemical phenotype included in our gene panel, the gene expression profile of manually harvested single neurons, that have undergone whole-cell recordings, closely match FACS-sorted single cells.

## Discussion

Underlying functionally discrete neuronal cell types in the brain are suites of differentially expressed genes that determine and maintain cell type identity (Nelson et al., 2006; Fishell and Heintz, 2013). An important category of these discriminatory genes are those that specify neurochemical phenotype, i.e. the expression of neuropeptides and markers of cholinergic, monoaminergic, purinergic and fast amino acid neurotransmission. In the hypothalamus, neuronal populations are often defined by their expression of neuropeptides, but an ever growing body of molecular, anatomic and functional evidence suggests that many hypothalamic neuronal cell types are equipped with a broader palette of neurochemical signaling mechanisms that may include multiple neuropeptides and fast neurotransmitters (Schöne and Burdakov, 2012; van den Pol, 2012; Arrigoni and Saper, 2014; Bonnavion et al., 2016). In the LHA, mounting evidence suggests that Hcrt/Ox and MCH neurons exhibit a complex but poorly understood repertoire of neuropeptide and fast neurotransmitter signaling mechanisms (Bonnavion et al., 2016). We undertook a quantitative single-cell gene expression profiling approach to better understand the neurochemical phenotype of Hcrt/Ox and MCH neurons. We optimized a robust procedure for microdissecting the LHA from transgenically-labeled mouse lines, cleanly isolating single Hcrt/Ox and MCH neurons via FACS and quantifying the expression of a panel of 48 genes in each cell to define neurochemical phenotype. We found that, in addition to expressing a number of neuropeptide transcripts, all MCH and a subpopulation of Hcrt/Ox neurons express markers for both inhibitory and excitatory fast neurotransmission. Furthermore, we found this profile is characteristic of a subpopulation of LHA glutamatergic neurons, but contrasts with a broad population of LHA GABAergic neurons. Finally, we found that electrical signatures that define Hcrt/Ox subpopulations do not appear to track with neurochemical phenotype.

### Comparison with previous approaches, technical considerations, and limitations

Previous studies of single-cell expression profiling in rodents were performed in rats; one probing the expression of five genes in manually harvested cells labeled with antibodies (Volgin et al., 2004) and another examining twelve genes from cells in fixed tissue, isolated using laser capture microdissection (LCM; Harthoorn et al., 2005). Two recent reports, however, describe single-cell microarray analysis (Liu et al., 2015) and RNA sequencing (Yelin-Bekerman et al., 2015) of zebrafish Hcrt neurons, which exhibit conserved function and expression of the key transcription factor gene *Lhx9*. Nevertheless, our approach represents an improvement over previous single-cell methods in the rodent LHA in terms of scale, throughput and precision. Furthermore, LCM has been shown to be significantly more prone to contamination, from glial cells in particular, as compared to FACS-sorted cells (Okaty et al., 2011). We found that robust expression of neuronal markers, with little to no expression of glial markers in these samples, was evidence for minimal contamination by glial cells. Furthermore, our failure to detect significant signal from single, fluorescent, FACS-sorted beads that were intermixed with our cell suspensions was evidence for minimal contamination by ambient transcripts in the media. With respect to the specificity of single-cell capture, we found that the vast majority of Hcrt/Ox and MCH neurons exhibited highly specific expression of their respective neuropeptides consistent with our demonstration that key transcription factor genes *Lhx9* and *Nkx2.1* exhibited selective and mutually exclusive expression.

However, the interpretation of our single-cell gene expression analysis must be considered in light of several important caveats. One is the specificity and penetrance of the mutant mouse lines that we used. Despite the presence of EYFP potentially reflecting both transient developmental and stable *Pmch* promoter-driven cre expression, *Pmch*-Cre;EYFP mice are very effective in selectively labeling *Pmch*-expressing neurons and capturing a large percentage of the MCH-IR population. Although Ox-EGFP mice show high specificity, the penetrance of their EGFP expression is comparatively low and we captured only about half of the Hcrt/Ox-IR population. Whether this represents a random cross-section of the Hcrt/Ox population or a specific, molecularly-defined subpopulation remains to be determined. Nevertheless, in each case, we independently assessed Hcrt/Ox or MCH expression through qPCR as an internal control to account for any lack of specificity of the transgenic line. Furthermore, the neurochemical profile of Hcrt/Ox neurons, that we determined through single-cell analysis, closely matched bulk RNA-TRAP data derived from another transgenic reporter with higher penetrance (Dalal et al., 2013; Fig. 4).

Another set of technical considerations in the interpretation of this data include: (1) the age and gender of the mice; (2) circadian, metabolic and behavioral state; (3) sample size; and (4) detection of mRNA as a reflection of translated protein. With regard to age, we chose to collect neurons from juvenile mice (P21-P23) to approximate baseline gene expression patterns in adult mice yet optimize the isolation of healthy and intact neurons following FACS sorting, which we found to be compromised in older animals. Although we chose to limit our study to males, future work comparing male and female mice will be required to probe possible sexual dimorphism in gene expression. In another effort to limit variability and obtain an approximation of baseline gene expression, we collected cells from mice at the same time of day in the absence of metabolic or behavioral challenges, which likely alters gene expression in LHA neurons (Leibowitz, 2007; Barson et al., 2013). Our qPCR and FISH data may be considered a snapshot of gene expression in juvenile male mice under these baseline conditions. With regard to sample size, we found that the number of single cells that we collected, along with minimal between-batch variability, had sufficient statistical power to detect even moderate levels of differential gene expression. However, further work, ideally employing whole transcriptome analysis of a large number of single neurons would be required to allow a higher resolution interrogation of neurochemical heterogeneity among these neurons. A final caveat in interpreting our single-cell qPCR data, as with any gene expression data, is that quantifying mRNA content with high sensitivity may or may not be a faithful, linear representation of translated protein (Citri et al., 2011). Also, given the stochastic nature of gene expression in single cells (Raj and van Oudenaarden, 2008), there may be an element of noise in the gene expression patterns that we observe, the extent to which, and biological significance of, remains to be determined.

### Neuropeptide/receptor coexpression in Hcrt/Ox and MCH neurons

Single MCH and Hcrt/Ox neurons express a variety of neuropeptide transcripts including some with clear differential expression. Hcrt/Ox neurons exhibit robust and selective expression of *Pdyn,* as shown previously (Chou et al., 2001; Muschamp et al., 2014), and moderately specific expression of *Penk*. MCH neurons, in turn, showed moderately specific expression of *Cartpt*, with apparent *Pmch*+ populations that were either *Cartpt*+ or *Cartpt*-, consistent with previous work (Broberger, 1999; Vrang et al., 1999; Brischoux et al., 2001; Elias et al., 2001; Cvetkovic et al., 2004), although we found more expression in Hcrt/Ox neurons than expected. Other transcripts (*Nucb2* and *Gal*) exhibit surprisingly abundant expression among the two populations. Previous studies showed strong colocalization between NUCB2-IR and MCH-IR but not with Hcrt/Ox-IR (Foo et al., 2008; Fort et al., 2008). While GAL-IR has been observed in Hcrt/Ox neurons (Håkansson et al., 1999), subsequent work showed no coexpression in Hcrt/Ox or MCH neurons (Laque et al., 2013). Despite the heterogeneity in neuropeptide transcript expression, clear subpopulations did not emerge from our unsupervised cluster analysis. Further work is required to determine if neuropeptide coexpression, or a constellation of other key markers, may be the primary driver(s) of population diversity among these and other LHA neurons.

Nevertheless, MCH and Hcrt/Ox neurons likely have the capacity to release multiple neuropeptides, evidenced by neuropeptide colocalization in single terminals (Guan et al., 2002; Balcita-Pedicino and Sesack, 2007) or even in the same dense-core vesicle (Muschamp et al., 2014). Furthermore, the release of multiple neuropeptides may significantly impact the ability of single neurons to tune their postsynaptic targets (van den Pol, 2012). For example, Hcrt/Ox and dynorphin cotransmission has been proposed to differentially regulate postsynaptic excitability among target neurons (Li and van den Pol, 2006; Arrigoni et al., 2010; Muschamp et al., 2014; Ferrari et al., 2015). Although Hcrt/Ox release from Hcrt/Ox fibers has been demonstrated using an optogenetic approach (Schöne et al., 2014), evoked release of multiple neuropeptides, and determining their role in circuit function, remains a significant technical challenge (van den Pol, 2012; Arrigoni and Saper, 2014).

### Fast neurotransmitter coexpression in MCH and Hcrt/Ox neurons

MCH neurons have long been thought to exhibit a GABAergic phenotype, evidenced by their expression of GABAergic markers such as GAD1 mRNA (Elias et al., 2001; Harthoorn et al., 2005; Sapin et al., 2010). Immunohistochemical evidence has shown that a small proportion of MCH+ varicosities found in the locus coeruleus (LC) contain VGAT and another small proportion oppose gephryin puncta on TH+ cell bodies (del Cid-Pellitero and Jones, 2012), suggesting the capacity of at least a small proportion of MCH neurons for synaptic GABA release. Consistent with these findings, optogenetic experiments showed that selective stimulation of MCH fibers evoked GABA release onto hypothalamic histaminergic (HA) neurons in brain slices (Jego et al., 2013). However, further work showed that MCH neurons may not fit the profile of canonical GABAergic neurons. First, MCH-IR was shown not to overlap with intermingled VGAT-expressing LHA neurons in *Vgat*-Cre;EYFP mice (Jennings et al., 2015). Furthermore, recent work shows that the vast majority of MCH neurons express mRNA for VGLUT2 while virtually none express VGAT, and that optogenetic activation of MCH fibers in the lateral septum induces monosynaptic release of glutamate, not GABA (Chee et al., 2015). The most parsimonious interpretation of our single-cell results is that MCH neurons are functionally glutamatergic (*Slc17a6*+) and uniformly capable of GABA synthesis (*Gad1*+), but that GABA release (*Slc32a1*-) is either mostly nonsynaptic or occurs through a noncanonical pathway as discussed further in the next section.

Convincing anatomic evidence suggests that Hcrt/Ox neurons are glutamatergic (Horvath et al., 1999; Guan et al., 2002; Rosin et al., 2003; Torrealba et al., 2003; Balcita-Pedicino and Sesack, 2007) including immunohistochemical evidence that a small proportion of Hcrt/Ox+ varicosities in the LC contain VGLUT2 and another small percentage oppose postsynaptic density protein-95 (PSD-95) puncta (Henny et al., 2010). Furthermore, optogenetic stimulation of Hcrt/Ox fibers induced glutamate release onto HA neurons (Schöne et al., 2012). As with MCH neurons, other data suggest ambiguity regarding the fast neurotransmitter phenotype of Hcrt/Ox neurons. Ultrastructural data in rats shows that while a majority of Hcrt/Ox terminals have asymmetric synapses, symmetric synapses are also present (Guan et al., 2002; Torrealba et al., 2003; Balcita-Pedicino and Sesack, 2007). Furthermore, recent work demonstrated that optogenetic activation of Hcrt/Ox neurons suppresses firing in nearby MCH neurons through light-evoked inhibitory synaptic events, which in some cases were time-locked to the stimulus with relatively short latencies, abolished by gabazine, but unaffected by glutamate receptor blockade. Although this may be explained by a disynaptic mechanism, GABA-IR was found in ∼10–20% of Hcrt/Ox-IR neurons (Apergis-Schoute et al., 2015). Our single-cell qPCR data are broadly consistent with the notion that Hcrt/Ox neurons are functionally glutamatergic (*Slc17a6*+), and that a subpopulation (∼50%) are potentially capable of GABA synthesis (*Gad1*+), but not release through a canonical pathway (*Slc32a1*-).

### Implications for neurochemical flexibility and plasticity among MCH and Hcrt/Ox neurons

We found that virtually all MCH neurons and approximately half of Hcrt/Ox neurons exhibit mixed fast neurotransmitter profiles (*Slc17a6+*, *Slc32a1-*, *Gad1+*). We also showed that this profile is characteristic of the broader population of LHA VGLUT2 neurons, half of which are *Gad1*+, suggesting that *Gad1* expression may not be a reliable marker for GABAergic phenotypes in this region. Formally, one explanation is that both MCH and Hcrt/Ox neuron populations have a functionally glutamatergic phenotype (*Slc17a6*+, *Slc32a1*-). Alternatively, given the prominent expression of *Gad1* among these neurons, it is tempting to speculate that MCH neurons and a subpopulation of Hcrt/Ox neurons may be capable of glutamate/GABA cotransmission through noncanonical GABA release pathways described in other systems (Seal and Edwards, 2006; Hnasko and Edwards, 2012; Münster-Wandowski et al., 2013; Barker et al., 2016; Tritsch et al., 2016; Granger et al., 2017). Anatomic and functional evidence for glutamate/GABA corelease from single neurons has been described in the developing auditory brainstem and among hippocampal mossy fibers during development and in the adult (Seal and Edwards, 2006; Hnasko and Edwards, 2012; Münster-Wandowski et al., 2013). Recent reports describe functional, monosynaptic glutamate/GABA cotransmission in basal ganglia outputs to the lateral habenula (LHb), with VGLUT2-IR and GAD-IR colocalized in terminals, as well as evidence that VGAT is necessary for cotransmission (Shabel et al., 2014). In addition, evidence for glutamate/GABA cotransmission was shown in two recent studies of ventral tegmental area (VTA) projections. In one, VGLUT2, VGAT and GAD appear to colocalize in single mesohabenular axons to facilitate glutamate/GABA corelease (Root et al., 2014). In the other, VGLUT2+ VTA neurons exhibit target-dependent monosynaptic cotransmission, with glutamate/GABA corelease in the LHb, but exclusive glutamate release in the nucleus accumbens (Yoo et al., 2016). In these latter cases, glutamate/GABA cotransmission appears to depend on colocalization of VGLUT2 and VGAT. However, among MCH and Hcrt/Ox neurons, *Slc32a1* mRNA was undetectable in our qPCR analysis and minimal in our FISH analysis. If MCH and a subset of Hcrt/Ox neurons are indeed capable of synaptic GABA corelease, it may occur through an alternative pathway. Noncanonical vesicular GABA release through VMAT2 (Tritsch et al., 2012) is an unlikely candidate in this case as *Slc18a2* is virtually undetectable among Hcrt/Ox and MCH neurons. Another alternative pathway is through reversal of the plasma membrane GABA transporter GAT-1 (Wu et al., 2007), which remains to be investigated. Finally, highly informative recent single-cell RNA sequencing studies of neuronal populations in the medial hypothalamus (Romanov et al., 2017; Campbell et al., 2017) and entopenduncular nucleus of the basal ganglia (Wallace et al., 2017) have revealed compelling evidence for mixed fast neurotransmitter gene expression profiles, suggesting that such forms of cotransmission may be more common than previously thought.

Regarding the *Gad1*+, *Slc32a1*- profile of MCH and Hcrt/Ox neurons, an informative comparison may be made with proopiomelanocortin (POMC) neurons in the arcuate nucleus. Detailed anatomic, electrophysiological and optogenetic analysis of the neurochemical phenotype of POMC neurons by Hentges and coworkers has revealed that this neuronal population may be parsed into two functional subpopulations: GABAergic (*Gad1+* and/or *Gad2+),* and a much smaller population of glutamatergic POMC neurons (*Slc17a6*+), half of which also express *Gad2* (Hentges et al., 2004, 2009; Jarvie and Hentges, 2012). Interestingly, POMC GABAergic neurons are capable of fast synaptic release of GABA (Hentges et al., 2009; Dicken et al., 2012) yet *Slc32a1* is virtually undetectable (Jarvie and Hentges, 2012), but may be expressed in small subpopulations (Jeong et al., 2016). Nevertheless, this suggests that GABA may largely be released from these cells through a noncanonical pathway. These same phenotypic markers were shown to change over postnatal development (Dennison et al., 2016) and can shift following metabolic perturbation (Dicken et al., 2015; Jarvie et al., 2017). Given evidence of alterations in neuropeptide gene expression in the LHA following metabolic and behavioral challenges (Leibowitz, 2007; Barson et al., 2013) and neurochemical phenotype switching in other systems (Demarque and Spitzer, 2012), it is plausible that MCH and Hcrt/Ox neurons also have the capacity for dynamic expression of key genes that specify neurochemical profile.

The possibility that MCH and/or Hcrt/Ox neurons exhibit neuropeptidergic and glutamate/GABA cotransmission, target-dependent cotransmission or the capacity for plasticity in neurotransmitter phenotype following metabolic or behavioral challenges all remain intriguing questions. Furthermore, the mechanisms of cotransmission and neurochemical heterogeneity may be the basis for functional classification of neuronal cell types in the LHA, as well as an important means of understanding their role in regulating postsynaptic excitability. Overall, our single-cell qPCR analysis of MCH and Hcrt/Ox neurons suggests the capacity for neurochemical flexibility: the ability to draw on a broad palette of signaling mechanisms with which to rapidly respond to fluctuating intrinsic and extrinsic physiologic challenges.

## Synthesis

Reviewing Editor: Lorna Role, Stony Brook Decisions are customarily a result of the Reviewing Editor and the peer reviewers coming together and discussing their recommendations until a consensus is reached. When revisions are invited, a fact-based synthesis statement explaining their decision and outlining what is needed to prepare a revision will be listed below. The following reviewer(s) agreed to reveal their identity: Ralph DiLeone, Young Hwan Jo.

The authors report their findings on the molecular properties of MCH and Hcrt/Ox neurons of the LHA obtained from gene mRNA expression profiling. The data provide insight into the extent of the heterogeneity of these neurons, and contribute to the complex literature on “mixed transmitter” phenotype of these neurons.

The delineation of the problem, experimental execution, and analysis are carefully completed and for the most part are very clearly presented. The manuscript is beautifully written and the documentation are clear.

However, after the reviewers and I had assessed the manuscript independently, we conferred and identified a few limitations of the current study. We have formulated specific questions about power sufficiency, the mouse lines that are being used for the isolation and analysis and confirmatory immunohistochemistry needed.

These are outlined in more detail below under major issues.

Note that we have specified how each issue must be addressed to render the manuscript apropos for eNeuro.

1. Is the Statistical Power sufficient with such a limited number of source animals?.

The qPCR work starts with mRNA obtained from five male Hcrt/Ox-marked and three male MCH-marked mice for the analysis presented here. For genomic analysis, this is problematic, as variation in a single animal could easily skew the data presented. There is significant concern on the part of both reviewers that with such a low number of source animals, the data may not be representative as it is subject to other effects not addressed here (e.g. litter effects). Using cells as the N for analysis obscures this potential problem and there is no clear attempt to address this.

There are several potential ways to address this issue.

The first is to process cells from more source animals (the reviewers felt that 7 animals/per line, if all male, would be optimal (=14 source animals; 7 per line which means adding 2 mice to the HCrt/Ox group and 4 mice to the MCH mice).

Ideally, since the study should compare males and females, the addition of more source animals in each sex would be ideal (e.g. 7 animals/ line /sex = 28 animals).

The authors should also provide an analysis of the variance of the data obtained from the current number of source animals, comparing the determinations by within animal and between animal groupings.

The actual number of source animals needed per line could then be addressed in a more thorough power analysis.

We do understand that if the authors have not collected cells from more animals that getting the n to sufficient power would likely extend the needed time beyond the 2 month limit. In this case, the authors should contact e Neuro and so that the return date/ status can be altered appropriately.

2. Verification of at least some of the identified PCR targets is needed.

Both reviewers felt that there is a need to validate at least some of the identified markers by immunostaining. In fact, MCH neurons appear to contain nesfatin 1, CART, galanin, proenkephalin, and orphanin FQ transcripts and orexin neurons express transcripts for prodynorphin, nesfatin 1, proenkephalin, etc. Expression of those transcripts does not mean that they indeed express those neuropeptides. Interestingly, all orexin neurons contain prodynorphin transcript.

At a minimum, the reviewers ask that confirmatory immunohistochemistry is done to address this question; i.e. immunostaining for (at least) two of the identified targets is essential ( e.g. immunohistochemistry for both orexin and dynorphin; are there no orexin only neurons?).

3. There are inherent limitations in using transgenic Cre driver lines.

As acknowledged and confirmed by the authors, the original Hcrt/Ox GFP line (Sakurai et al., 1999) only labels a subset of Hcrt/Ox neurons. As a goal of the present study is to understand the heterogeneity of Hcrt/Ox neurons, using a line in which only a subset of the neurons are labeled (reported as ~15% of Hcrt/Ox neurons in other studies using this line), is, at best, problematic as the authors do not understand what subset of Hcrt/Ox neurons are labeling. The MCH labeled line has the opposite problem- neurons that are labeled that clearly do not express MCH.

While the authors note these issue, this suboptimal “input” nonetheless limits the breadth and impact of the analysis. Correlations with previous studies were completed on subset (41) of “known discriminatory” genes, but with moderate results in this selective context (0.71 r value with p value unclear). At an absolute minimum these clear limitations of the study must be explicitly delineated in the Discussion.

While overall, the Discussion is impressive and scholarly and reflects the overall effort with the preparation of this manuscript, there is a notable absence of consideration of the limitations of the work.

Minor concern;

1. A recent study (Jeong et al., 2016) shows that ARC POMC neurons express vesicular GABA transporter (Slc32a1), so the authors need to correct the sentence (line 750-751) in the discussion.
